# Comparison of Magnetic Deflection among Neutral Sodium-Doped Clusters: Na(H_2_O)*_n_*, Na(NH_3_)*_n_*, Na(MeOH)*_n_*, and Na(DME)*_n_*

**DOI:** 10.1021/acs.jpca.3c03820

**Published:** 2023-10-05

**Authors:** Jonathan V. Barnes, Dominique P. Borgeaud dit Avocat, Edith Simmen, Huanyu Yang, Bruce L. Yoder, Ruth Signorell

**Affiliations:** Department of Chemistry and Applied Biosciences, ETH Zürich, Vladimir-Prelog-Weg 2, Zürich 8093, Switzerland

## Abstract

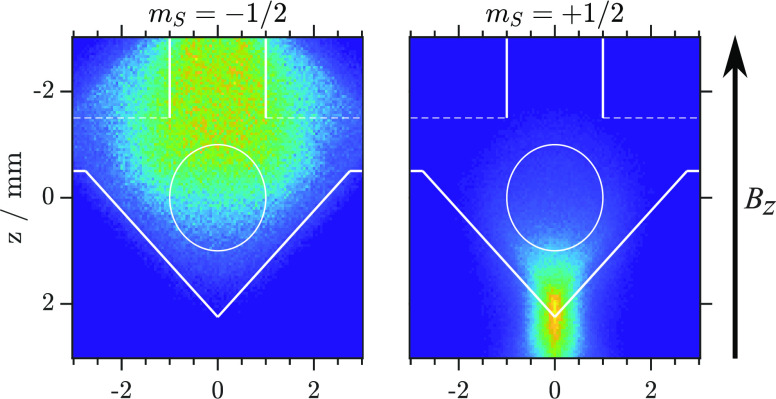

Using a pulsed Stern–Gerlach deflection experiment, we present the results of a comparative study on the magnetic properties of neutral sodium-doped solvent clusters Na(Sol)*_n_* with *n* = 1–4 (Sol: H_2_O, NH_3_, CH_3_OH, CH_3_OCH_3_). Experimental deflection ratios are compared with values calculated from molecular dynamics simulations. NaNH_3_ and NaH_2_O are deflected as a spin 1/2 system, consistent with spin transitions occurring on a time scale significantly longer than 100 μs. For all other clusters, reduced deflection is observed. The observed magnetic deflection behavior is correlated to the number of thermally populated rotational states in the clusters. We discuss that spin–rotational couplings allow for avoided crossings and a reduction in the effective magnetic moment of the cluster. This work attempts to understand the evolution of magnetic properties in isolated weakly bound clusters and is relevant to diamagnetic and paramagnetic species expected to exist in solvated electron systems.

## 1 Introduction

Alkali metal-doped solvent clusters provide ideal experimental and theoretical model systems to study properties of excess electrons as a function of system size^1−13^ (and references therein). The formation of solvated, spin-paired electrons as a function of alkali metal concentration in sodium-doped nanodroplets^14,15^ and liquid bulk solutions^16^ is of current interest. Probing spin-pairing effects via photoelectron spectroscopy (PES) is limited in its application due to the similar electron kinetic energies of the involved species. However, investigating differences in the magnetic properties of sodium-doped clusters might be a favorable approach to probe spin-pairing effects. Distinguishing diamagnetic (singlet) and paramagnetic (triplet) states of singly sodium-doped clusters using Stern–Gerlach (SG) deflection is suggested here as a first experimental step toward future studies of more complex systems.

The original SG experiment was designed to determine the quantization of the electron spin in silver atoms.^17,18^ In atoms, the total angular momentum *J* is composed of the orbital angular momentum *L* and its intrinsic spin angular momentum *S*, neglecting the nuclear spin. In an SG experiment, each Zeeman level corresponds to an individual deflecting beamlet.^19^ In polyatomic molecules, additional degrees of freedom may contribute to the total angular momentum. The molecular Zeeman effect is analogous to that in atoms, with additional contributions of angular momenta (e.g., rotational and vibrational). Theoretical investigations of the diatomic case were performed by Hill^20^ and later Schadee.^21^ An overview of the theory in diatomic molecules is given by Berdyugina and Solanki,^22^ where analytical expressions of the Zeeman levels are given. Gedanken et al.^23,24^ showed the importance of spin–rotation interaction in SG deflection experiments of oxygen and nitrogen oxide radicals.

Amirav and Navon^25,26^ found that paramagnetic molecules and stable radicals (TEMPO: 2,2,6,6-tetramethylpiperidine-*N*-oxyl and DTBN: di-*tert*-butyl nitroxide) were deflected less than predicted. They attributed the discrepancy between experiment and prediction to be due to fast intramolecular spin relaxation induced by spin–orbit coupling, causing a loss of orientation of the magnetic moment and reduced deflection. However, Gedanken et al.^23^ found that magnetic deflection spectra of TEMPO combined with line-profile calculations did not support the interpretation of spin relaxation processes occurring while traversing the deflector. For large paramagnetic molecular and cluster systems, the density of Zeeman-like levels becomes so high that quantum chemical calculations are prohibitively expensive. Various groups^27−29^ developed more simplified theoretical models to explain SG deflection experiments within terms of intramolecular spin relaxation effects caused by several spin transitions while conserving the total angular momentum. De Heer and co-workers^30−33^ observed one-sided deflection for Fe_*m*_, Co_*m*_ and Ni_*m*_ clusters (*m* = 10–1000), which was interpreted as a rapid intracluster spin relaxation (ISR) process.^27^ Recent studies show similar deflection behavior for metal–organic sandwich clusters^34−37^ and paramagnetic superatoms.^38−41^

Here, we employ an SG deflection experiment to study the magnetic properties of neutral sodium-doped solvent cluster Na(Sol)*_n_* with 1 ≤ *n* ≤ 4 (Sol: H_2_O, NH_3_, CH_3_OH, CH_3_OCH_3_). Interpretation of the SG deflection of molecular clusters is still a challenge. Singly sodium-doped solvent clusters are promising model systems as several characteristic properties can be exploited in order to understand their magnetic properties as a function of system size and type of solvent molecules. In the case of singly sodium-doped clusters with *m_s_* = ±1/2, zero field splitting terms are vanishing, and high-symmetry highest occupied molecular orbitals (HOMOs) suggest that contributions of spin–orbit coupling may be minor in some cases.^10,11^ In the presented work, hyperfine effects from couplings to nuclear spins are neglected, although Fuchs et al.^42^ demonstrated that nuclear spin can diminish electron spin coherence in metal clusters. The magnetic deflection behavior of various sodium-doped clusters is discussed in terms of thermally accessible rovibrational states. Spin–rotational couplings can produce avoided crossings between Zeeman-like levels of the same *J*. We observe that the SG deflection behavior of the studied clusters appears to be linked to thermally accessible rovibrational states and that the measured magnetic deflection can be inversely related to the density of the clusters’ rotational state manifolds.

## 2 Methods

### 2.1 Experimental Setup

**Figure 1:**
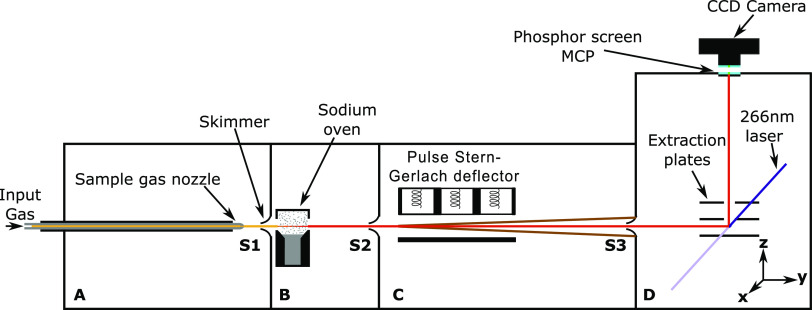
Sketch of the experimental setup consisting of the source chamber (A), sodium oven chamber (B), deflection chamber (C), and detection chamber (D). For a detailed description see text below and ref (43).

The experimental setup to study size-dependent magnetic properties of neutral sodium-doped solvent clusters has been previously described in detail.^43^ Nevertheless, a short description of the experimental and theoretical methods is given here. A sketch of the experimental setup is shown in Figure 1.

Molecular clusters are generated via supersonic expansion of either neat gas or mixtures with He or N_2_ into a vacuum (A). The cluster formation conditions of the individual substances are summarized in Table S1 of the Supporting Information (SI). The expansion is skimmed at S1, and the resulting cluster beam was doped with sodium as it traverses the temperature-controlled oven in the chamber (B). See Table S1 for the Na-oven temperatures used in the presented work (SI).

The sodium-doped solvent clusters pass a 1.5 mm diameter skimmer (S2) to enter the deflection chamber (C) which houses the pulsed SG deflector. The magnetic field gradient d*B*/d*z* in the deflector flight channel acts on the paramagnetic clusters, causing the cluster beam to diverge. We have included a description of the magnetic field pulses in the SI. The magnetic field gradient obtained from COMSOL simulations at 1000 A deflector current is shown in Figure 1d in ref (43) and in the SI. Selection of clusters after the SG deflector is performed by a 2 mm diameter skimmer (S3) at the entrance of the detection chamber (D). With the existing experimental setup, we are able to measure clusters that are transmitted only through S3.

Upon reaching the ionization region inside the extraction optics, the neutral clusters are ionized with a pulsed (∼7 ns) 266 nm (4.66 eV) Nd:YAG laser. The resulting photoions are extracted perpendicular to the molecular beam axis and detected by either time-of-flight (TOF) mass spectrometry or velocity map imaging (VMI). Ion-resolved VMI was achieved by gating the microchannel plates of the imaging detector to only be high when the ions of interest arrived at the detector. The perpendicular configuration of the molecular beam axis and TOF axis allows us to determine molecular beam velocities *v*_*y*_. The displacement from the image center *r* is related to the neutral clusters’ velocity *v*_*y*_ by
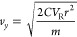
1where *V*_R_ is the voltage on the repeller, *m* is the cluster mass, and *C* is a setup-dependent calibration constant. Velocity distributions of the sodium-doped solvent clusters obtained in this study are listed in Section 3.

Highest possible deflection in our pulsed SG setup is achieved by synchronization of magnetic field pulse trigger timings of the three coils in the deflector setup *t*_1_, *t*_2_, and *t*_3_ to the laser timing *t*_L_. These relative time delays are optimized to the velocity distribution of each cluster beam studied (see ref (43)) in order to achieve optimal deflection. Extracting photoions in VMI conditions and mass-gating allow us to record velocity-dependent and cluster-resolved deflection data. The relative signal of the photoions θ_rel_ is defined as the ratio of the signal with the deflector “on”, θ_on_, and the deflector “off”, θ_off_.
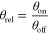
2Evaluating θ_rel_ as a function of the relative deflector delay *t*_d_ is characterized by a distinct signal minimum. The signal minimum is quantified as the deflection ratio γ_d_ for various deflector currents *I*_d_.
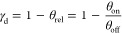
3where γ_d_ = 1 indicates that 100% of particles are deflected and corresponds to θ_on_ = 0.

We do not expect fragmentation or monomer evaporation of these weakly bound clusters to be significant upon photoionization. We are ionizing the Na atom and not the monomer units of the cluster, and the outgoing photoelectron is carrying away the excess energy of the photon as the kinetic energy. Na-doping and UV ionization has been established as an ultrasoft method for cluster size determination.^44,45^ Furthermore, as an experimental verification that the magnetic deflector did not alter the cluster signal, we collect mass spectra and VMI’s with the deflector “off” and with the deflector “on” but with the ionizing laser pulse not synchronized to interest the clusters that experienced the magnetic field. The signals showed no significant deviations.

### 2.2 Modeling of the Deflection

A detailed description of the molecular dynamics (MD) approach to model the deflection process has been given previously.^43^ Here, we summarize the main aspects of our approach and point out improvements from earlier work. Cluster trajectories are simulated from the entry of the deflector to the ionization region. The initial coordinates (*x*, *y*, *z*) and momentum (*p*_*x*_, *p*_*y*_, *p*_*z*_) of each particle are defined in the cluster beam, where the mass is defined as a given cluster mass. The velocity in the molecular beam direction (*y*-axis; see Figure 1) is sampled from experimentally determined velocity distributions. The velocity components in the *x*, *z*-direction are calculated from the beam divergence angle and *v*_*y*_. More than 10^5^ particle trajectories are typically simulated. The experimental dimensions of the deflector, flight distances, skimmer diameters, and positions, as well as the ionization region, are used in our model. For particles which reach the ionization region simultaneously at time *t*_L_, an output is written. The simulation output consists of coordinates (*x*_*i*_, *y*_*i*_, *z*_*i*_) at ionization and corresponding momentum (*p*_*x*_, *p*_*y*_, *p*_*z*_) for particles of spin states −1/2, 0, and +1/2. Simulations of *S* = 0 produce results equivalent to those of deflector “off”. Instead of taking the topology of the rovibrational Zeeman diagram into account to simulate the magnetic deflection behavior of the clusters, we have introduced an optional additional scaling factor of the magnetic moment to account for possible ISR effects. We scale the magnetic moment μ_0_ of a single *m*_*s*_ = ±1/2 particle with an exponential decay, defined by the interaction time with the magnetic field *t*_m_ and a characteristic relaxation time τ.
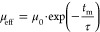
4
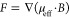
5where *t*_m_ = *s*_def_/*v*_*y*_ is defined by the deflector length *s*_def_ and molecular beam velocity *v*_*y*_. By sampling various values of τ and comparing simulations to experimental deflection results, we estimate the characteristic spin relaxation times τ for the investigated clusters. With modeled effective magnetic moments μ_eff_, we quantitatively compare the deflection behavior of the clusters.

## 3 Results

### 3.1 Magnetic Deflection of Na(H_2_O)*_n_*

#### 3.1.1 NaH_2_O

**Figure 2:**
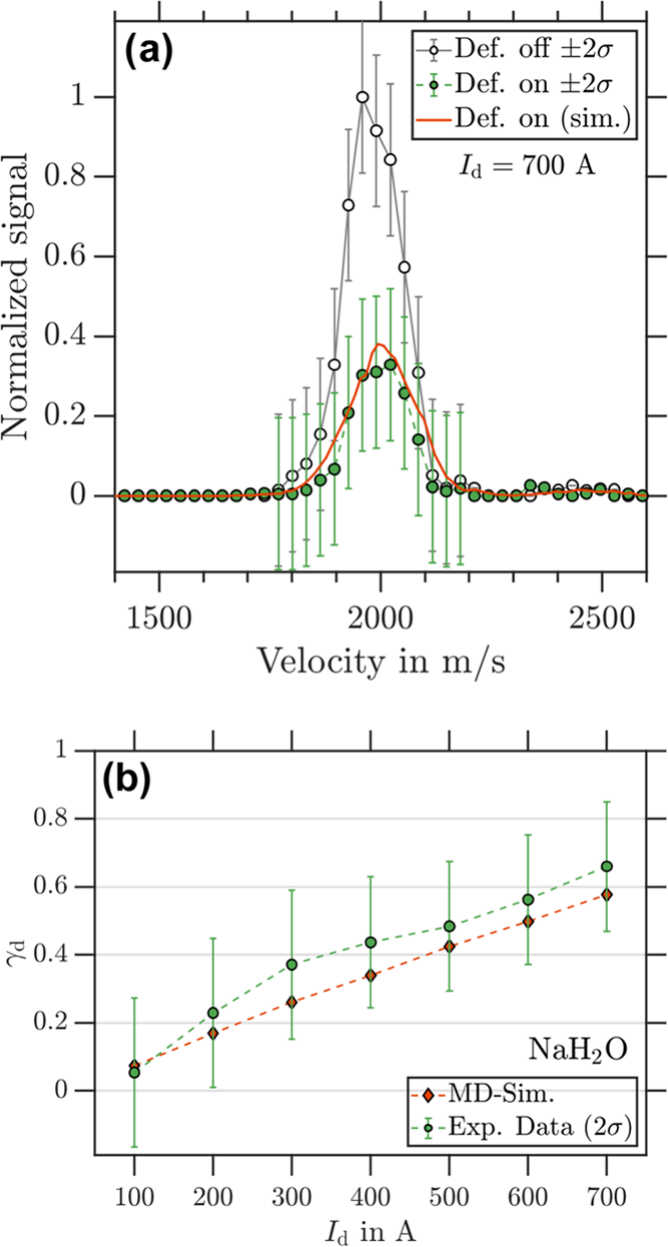
(a) Experimental NaH_2_O velocity distribution retrieved from photoion VMI for deflector “off” (black circles) and deflector “on” (green dots) at *I*_d_ = 700 A. The corresponding simulated NaH_2_O velocity distribution for *m_s_* = ±1/2 particles are shown as red traces. (b) γ_d_ as a function of *I*_d_ for NaH_2_O with experimental data in green and simulation data in red.

Figure 2a shows the NaH_2_O velocity distributions (obtained from VMIs) for deflector “off” and “on” measurements at an operating current of 700 A and optimized deflector coil timings^43^ for the sampled velocity distribution (center velocity *v*_c_ = 2000 m/s, full width at half-maximum (fwhm) = 150 m/s) to achieve maximal possible deflection. Under these conditions, we measured a deflection ratio, γ_d_, of 0.66(10) as shown in Figure 2b. Agreement between the experiment and simulations is found across the velocity distribution within experimental errors. Figure 2b summarizes the experimental γ_d_ values for all sampled *I*_d_. Deflection (γ_d_ > 0) is observed for *I*_d_ ≥ 200 A and agrees with the MD simulations for each sampled deflector current. We note that the experimental γ_d_ is slightly and systematically higher than the simulations for a spin 1/2 particle and suspect collisions of the cluster beam with background gas molecules (e.g., outgassing from the deflector) while the deflector is switched “on” to be a possible explanation for the systematic deviations. The heating of the deflector arises from thermal loads generated by the in-vacuum coils that cannot be completely dissipated with our cooling system. A total loss of the signal occurs at a chamber pressure above 5 × 10^–4^ mbar, which we attribute to obstruction of the flight channel (see the SI of ref (43)). The additional signal depletion contributes to measured deflection due to the comparison to deflector “off” measurements. Within experimental error, the deflection behavior of NaH_2_O is equal to that of an *m_s_* = ±1/2 particle. As we are only sensitive to processes occurring on time scales that are either faster or similar to *t*_m_, we have the condition that τ > *t*_m_ must hold in order for a cluster to behave like an *m_s_* = ±1/2 particle in our experiment. For the data shown in Figure 2, *t*_m_ is ∼100 μs, which is consistent with τ > 100 μs for NaH_2_O.

In a previous study, we observed deflection behavior of an *m_s_* = ±1/2 particle for NaNH_3_ and reduced deflection for cluster sizes of *n* > 1.^43^ This raises the question whether Na(H_2_O)*_n_* clusters with *n* > 1 exhibit a similar cluster size dependence in magnetic deflection.

#### 3.1.2 Na(H_2_O)_2–4_

**Figure 3:**
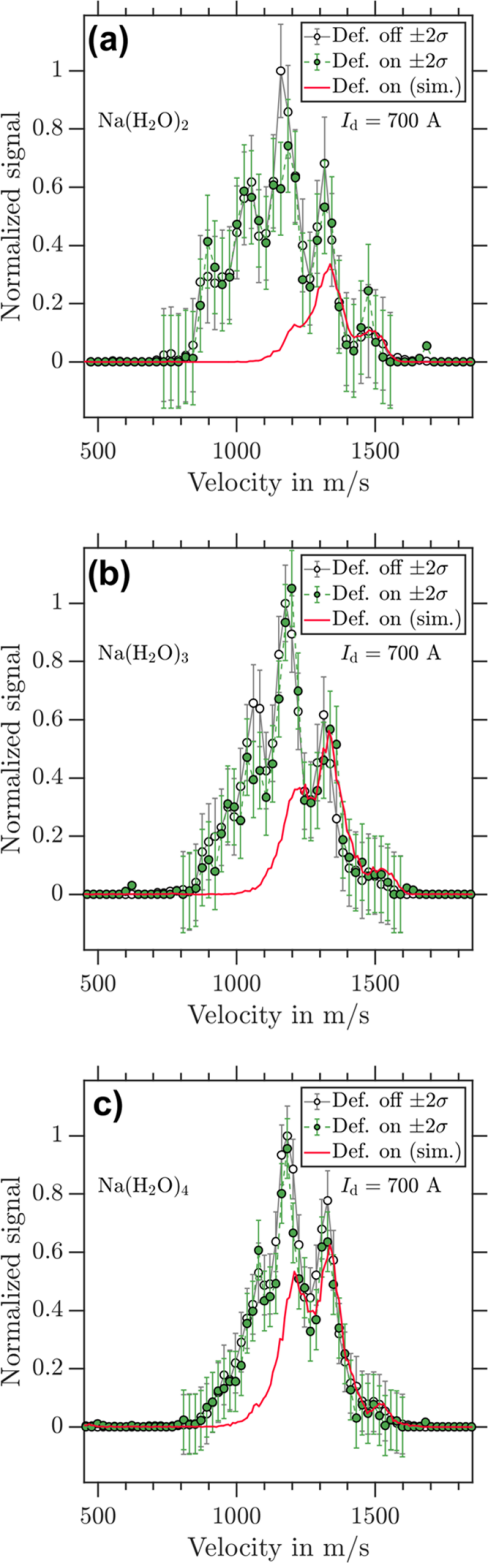
Experimental velocity distributions of (a) Na(H_2_O)_2_, (b) Na(H_2_O)_3_, and (c) Na(H_2_O)_4_ retrieved from photoion VMIs for deflector “off” (black circles) and deflector “on” (green dots) at *I*_d_ = 700 A. The corresponding simulated velocity distributions for *m_s_* = ±1/2 are shown as red traces.

The deflection results for the highest possible magnetic field gradient (*I*_d_ = 700 A) and a broad velocity distribution of the cluster beam (∼800–1500 m/s) are shown in Figure 3 for (a) Na(H_2_O)_2_, (b) Na(H_2_O)_3_, and (c) Na(H_2_O)_4_. The distinct peaks of the velocity profiles are caused by mechanical recoils of the pulsed Even–Lavie valve^46^ used to generate the beam of water clusters. The simulations predict full deflection (θ_rel_ = 0) for velocities <1100 m/s. This, however, is not observed in the experiment and means that these clusters do not interact with the magnetic field as free spin 1/2 systems. The results for Na(H_2_O)_2_, Na(H_2_O)_3_, and Na(H_2_O)_4_ are consistent with spin transitions taking place on significantly faster time scales than the experiment. In the simulated data for a spin 1/2 particle (red traces) of Figure 3, the residual signal to the fast side of the velocity distribution results from the magnetic field pulses being too short in time to act on all of the velocities present in the molecular beam. The pulses of the magnetic field were optimized to deflect the slower velocities but were not long enough in time to act on all velocity components present in the cluster beam. This can be seen by the simulated data for a spin 1/2 particle being fully deflected for the slower velocities, partially deflected for the intermediate, and nondeflected for the fastest velocities of the profile in Figure 3.

### 3.2 Magnetic Deflection of Na(MeOH)*_n_*

#### 3.2.1 NaMeOH

**Figure 4:**
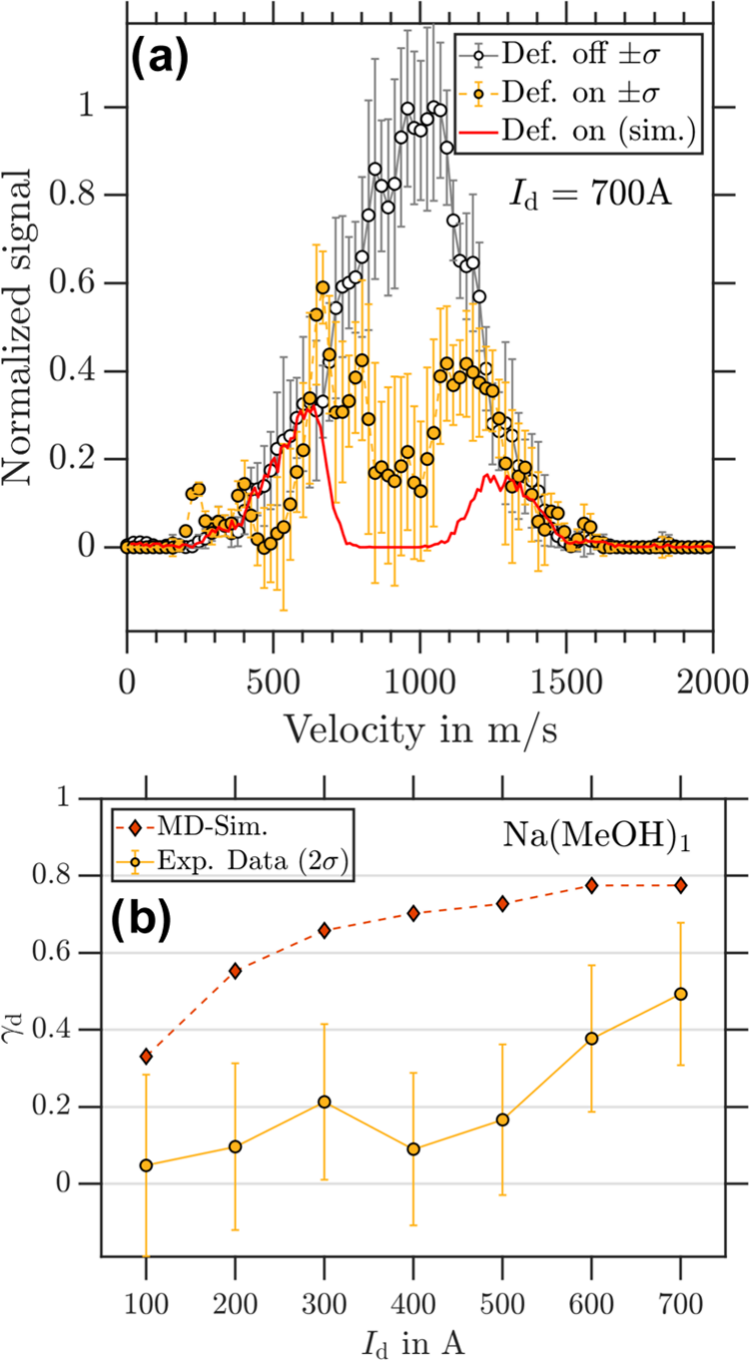
(a) Experimental NaMeOH velocity distributions retrieved from photoion VMIs for deflector “off” (black circles) and deflector “on” (yellow dots) with *I*_d_ = 700 A. The corresponding simulated velocity distributions for *m*_*s*_ = ±1/2 particles obtained from simulations are shown as red traces. (b) γ_d_ as a function of *I*_d_ for NaMeOH with experimental data in yellow and simulation data in red.

Figure 4a displays velocity-calibrated photoion VMIs of NaMeOH for deflector “off” and deflector “on” measurements at *I*_d_ = 700 A. NaMeOH exhibits deflection with θ_rel_ = 0.5(1) over the full velocity distribution, and minimal relative signal (θ_rel_ = 0.2(1)) in the velocity range of 800–1100 m/s. The residual signals to the slow and fast side of the velocity distribution are a result of the applied magnetic field pulses, which are too short in time to ensure significant magnetic field gradients across the sampled velocity distribution. For the data shown in Figure 4a, the magnetic field pulse timings were optimized for deflection of velocities near the center of the molecular beam’s velocity profile. The slowest and fastest particles of the molecular beam did not experience a significant magnetic field gradient, and their trajectories were left unperturbed. Although NaMeOH exhibits clear deflection, it is less than the simulations predicted for an *m*_*s*_ = ±1/2 system. Figure 4b depicts γ_d_ as a function of the deflector current, where partial deflection is observed for *I*_d_ > 500 A. Again, the MD simulations are not in agreement with the experimental observations.

#### 3.2.2 Na(MeOH)_2–4_

**Figure 5:**
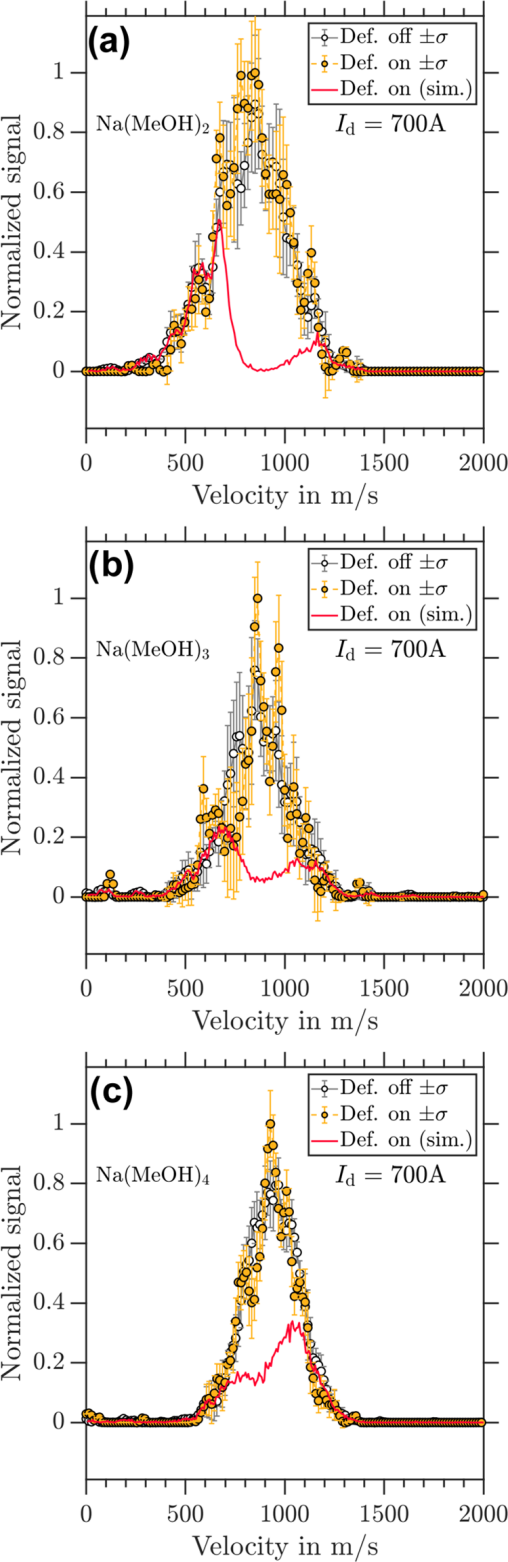
Experimental velocity distributions of (a) Na(MeOH)_2_, (b) Na(MeOH)_3_, and (c) Na(MeOH)_4_ retrieved from photoion VMIs for deflector “off” (black circles) and deflector “on” (yellow dots) at *I*_d_ = 700 A. The corresponding simulated velocity distributions for *m_s_* = ±1/2 are shown as red traces.

The deflection results for *I*_d_ = 700 A are shown in Figure 5 for the clusters (a) Na(MeOH)_2_, (b) Na(MeOH)_3_, and (c) Na(MeOH)_4_. None of these clusters exhibits deflection across their sampled velocity distributions. The deflector “off” trace and deflector “on” traces are the same within experimental error. The obtained simulations, however, predict deflection for all three cluster sizes. These deviations of experiment and simulation again show that the magnetic properties of the investigated clusters cannot be described by the magnetic moment of *m_s_* = ±1/2 particles.

For Na(NH_3_)*_n_* (see ref (43)) and Na(H_2_O)*_n_* (see Section 3.1), we noted that an increase in cluster size, *n*, was correlated with a decrease in the experimentally observed deflection, γ_d_. Similar cluster-size-dependent deflection behavior is observed for Na(MeOH)*_n_*. The presented results can be explained by ISR processes taking place on significantly faster time scales than the experiment. In order to discuss possible ISR processes of partially and nondeflecting clusters, we analyze thermally accessible rovibrational states as a function of cluster size, *n* see (Section 3.5).

### 3.3 Magnetic Deflection of Na(DME)*_n_*

#### 3.3.1 NaDME

**Figure 6:**
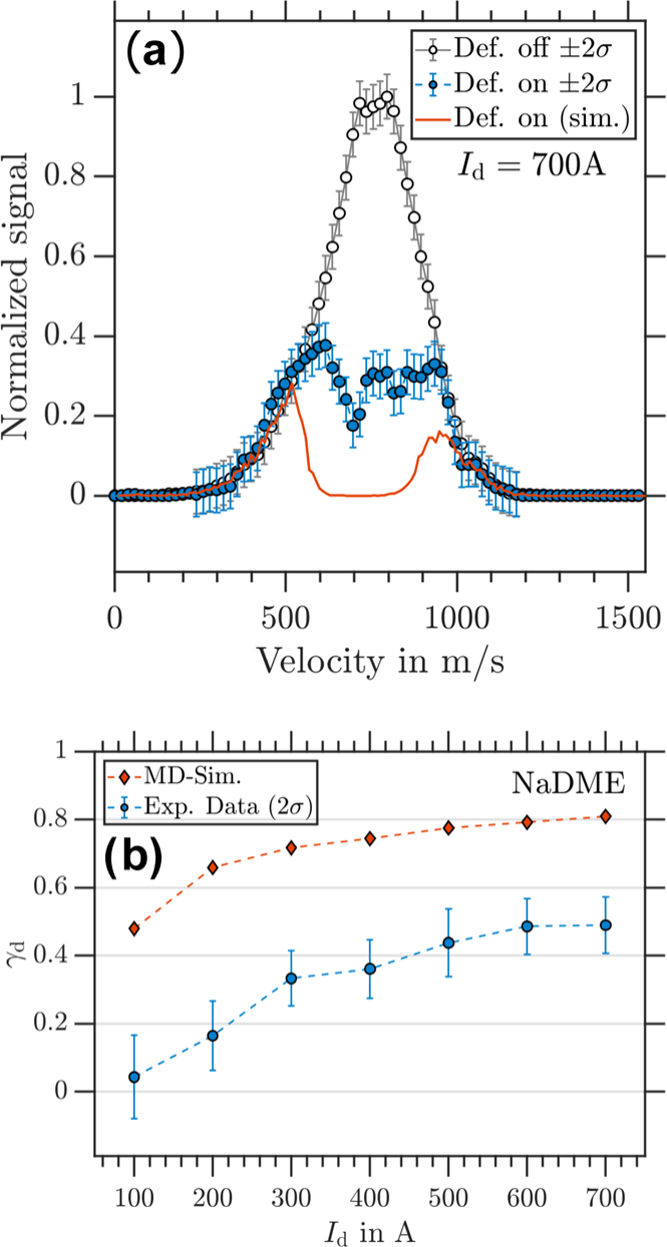
(a) Experimental NaDME velocity distribution retrieved from photoion VMI for deflector “off” (black circles) and deflector “on” (blue dots) at *I*_d_ = 700 A. The corresponding simulated NaDME velocity distribution for *m*_*s*_ = ±1/2 particles is shown by the red trace. (b) γ_d_ as a function of *I*_d_ for NaDME with experimental data in blue and simulation data in red.

Velocity-calibrated photoion VMIs of NaDME are shown in Figure 6a for deflector “off” and “on” measurements at *I*_d_ = 700 A. We determined θ_rel_ = 0.49(5) for the sampled velocity distribution. The corresponding simulated data exhibits full deflection (θ_rel_ = 0) for velocities between 600 and 900 m/s, but incomplete deflection (θ_rel_ ≈ 0.20) for slower ≤600 m/s and faster ≥900 m/s particles. However, the simulation did not match our experimental observation. Figure 4b shows γ_d_ as a function of deflector current, where deflection is observed for *I*_d_ > 100 A. Quantitative agreement between experiment and simulation is not found for any of the studied deflector currents, although deflection increases with increasing deflector current in both.

For NaDME, the deviation between simulations and experiment may be due to ISR processes, which occur on similar time scales to the interaction time with the magnetic field, causing a reduction of the cluster’s magnetic moment.

#### 3.3.2 Na(DME)_2_

**Figure 7:**
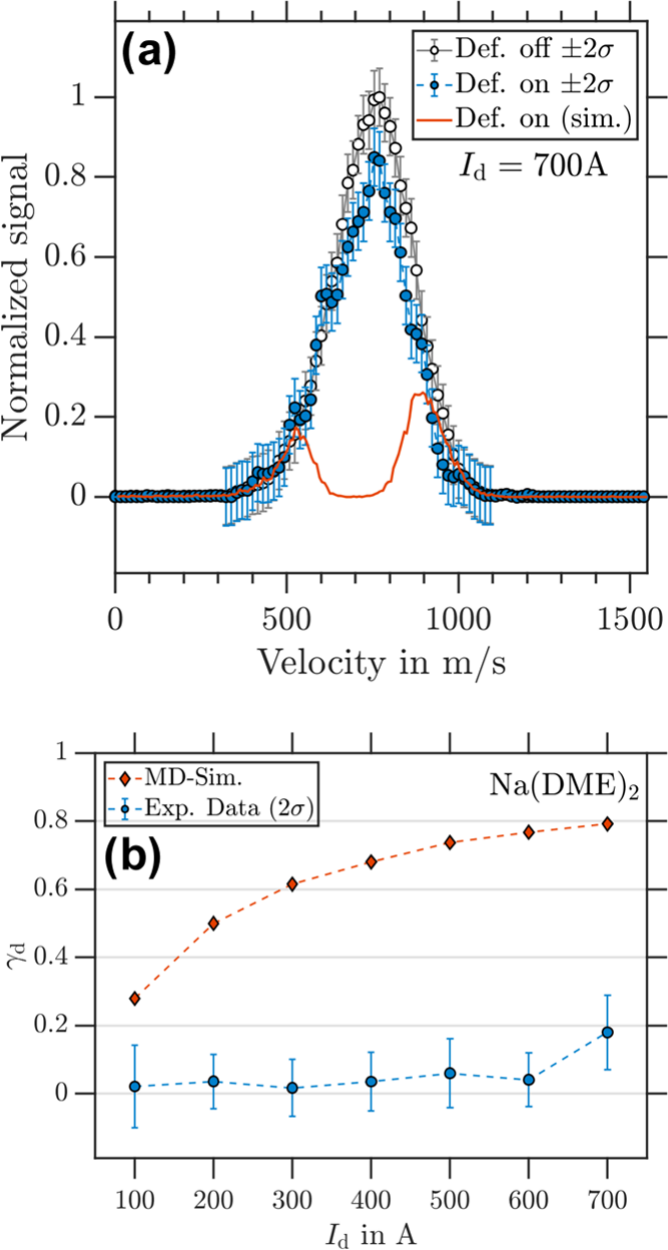
(a) Experimental Na(DME)_2_ velocity distribution retrieved from photoion VMIs for deflector “off” (black circles) and deflector “on” (blue dots) at *I*_d_ = 700 A. The corresponding simulated Na(DME)_2_ velocity distribution for *m_s_* = ±1/2 particles is shown as red traces. (b) γ_d_ as a function of *I*_d_ for Na(DME)_2_ with experimental data in blue and simulation data in red.

Magnetic deflection data of Na(DME)_2_, analogous to that of NaDME, is presented in Figure 7. At *I*_d_ = 700 A (Figure 7a), the deflector “on” signal is slightly decreased when compared to the deflector “off” signal, which results in an overall θ_rel_ = 0.82(7). According to the simulations, full deflection is expected in the velocity range of 600–800 m/s and residual signals for slower ≤600 m/s and faster ≥800 m/s particles. We classify Na(DME)_2_ as partially deflected at *I*_d_ = 700 A, whereas at lower deflector currents, no significant deflection was observed (see Figure 7b).

The reduced deflection observed for Na(DME)_2_ may be due spin transitions occurring on a time scale similar to the interaction time with the magnetic field gradient. It is interesting to note that deflection of Na(DME)_2_ becomes observable at *I*_d_ > 600 A. The higher deflector current would produce a stronger magnetic field gradient and a larger Zeeman splitting, which could make more avoided crossings of Zeeman-like states. For *I*_d_ > 600 A, it appears that the magnetic field gradient is large enough to deflect some Na(DME)_2_ clusters, even though we essentially determine μ_eff_ = 0 at lower currents.

#### 3.3.3 Na(DME)_3_

**Figure 8:**
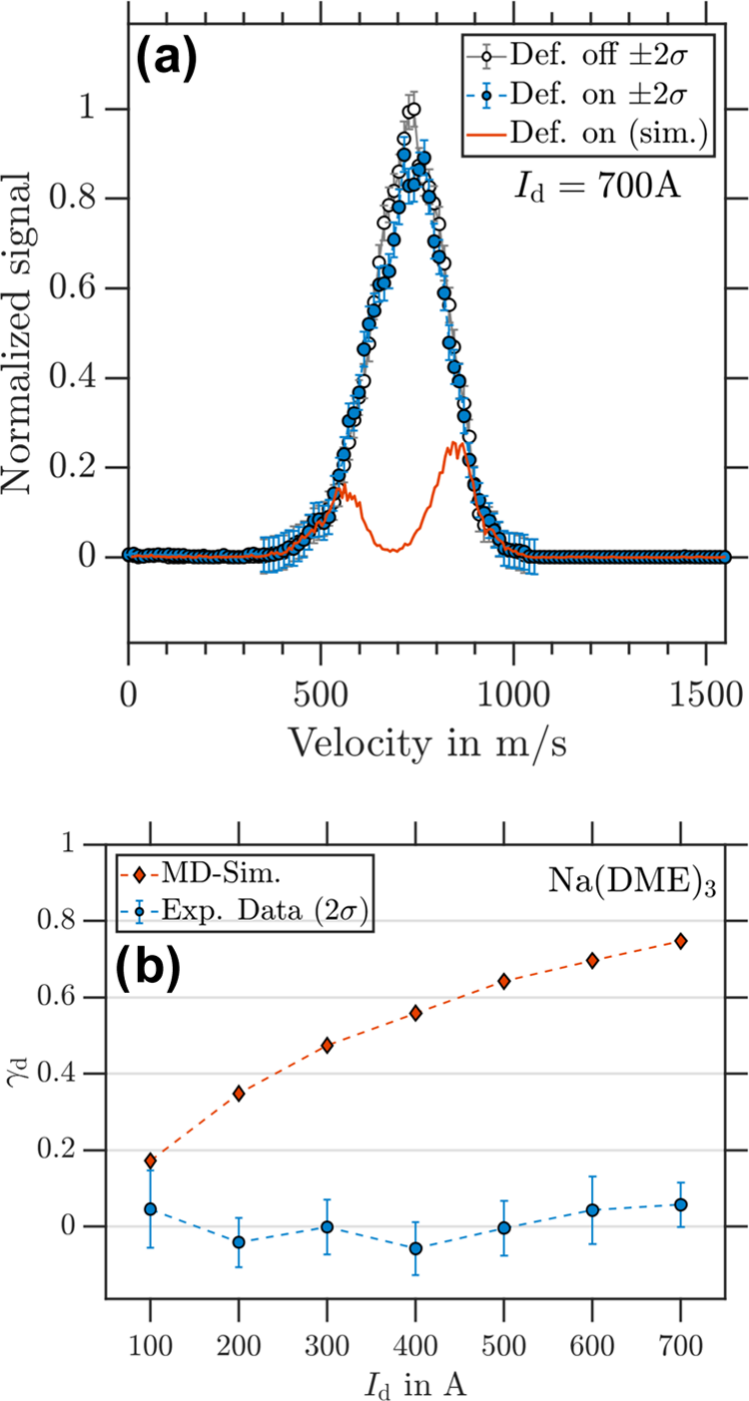
(a) Experimental Na(DME)_3_ velocity distribution retrieved from photoion VMI for deflector “off” (black circles) and deflector “on” (blue dots) at *I*_d_ = 700 A. The corresponding simulated Na(DME)_3_ velocity distribution for *m_s_* = ±1/2 particles is shown with the red trace. (b) γ_d_ as a function of *I*_d_ for Na(DME)_3_ with experimental data in blue and simulation data in red.

Calibrated photoion VMIs of Na(DME)_3_ at *I*_d_ = 700 A are shown in Figure 8a. We classify Na(DME)_3_ as nondeflected, although slight deflection might be visible in the region of the center velocities (650–850 m/s). We needed increased sensitivity to make a definitive statement. The simulated signal in contrast reduces to θ_rel_ = 0.25 and as in the case of previous clusters exhibiting reduced deflection. Again, the deflection process is not described by the interaction of *m*_*s*_ = ±1/2 particles with the inhomogeneous magnetic field. Measurements at lower deflector currents confirm this deflection behavior since γ_d_ = 0 was measured (see Figure 8b).

The differences between experiment and simulation found for Na(DME)*_n_* (*n* = 1–3) are discussed in terms of possible ISR processes as a function of cluster size *n* in Section 3.5.

### 3.4 Modeling of an Effective Magnetic Moment

The effective magnetic moments μ_eff_ of NaH_2_O (see Section 3.1.1) and NaNH_3_ (see ref (43)) are equal to the magnetic moment μ_0_ of an *m*_s_ = ±1/2 particle within our experimental uncertainty. All other clusters can be described with μ_eff_*<* μ_0_. As previously mentioned, it is expected that ISR processes would result in a reduction of μ_0_. By introducing an attenuation factor *a*, we describe the simulated deflection with μ_eff_ = μ_0_·*a*. It has been discussed^27,29,47^ that ISR processes are determined by the topology of the Zeeman diagram, the thermal population of spin–rotational-coupled Zeeman-like levels, spin–rotational coupling constants, and the magnetic field gradient ∇*B* a cluster experiences while traversing the deflector.^27,29,47^ An exact description considering all relevant interactions in detail is a complex task, and we therefore choose a simplified model to account for spin relaxation effects. Modeling the effective magnetic moment via the Curl formula^48^ may be a valid approach for cluster systems which behave as rigid rotors with only a few vibrational states populated. Recently, Rivic et al.^49^ have elegantly demonstrated this.

As stated in eq 4, we assume an exponential decay of μ_0_ during the transit time *t*_m_ with a characteristic relaxation time τ. Performing MD simulations with μ_eff_ for various characteristic relaxation times τ (25, 50, 75, 100, 125, and 150 μs) allows us to match the simulations to our experimental observations. For each τ value, the squared deviations (γ_d,sim_ – γ_d,exp_)^2^ are evaluated by their χ^2^ value given by
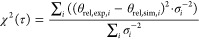
6Here, σ*_i_* is the experimental error of each sampled velocity *v*_*i*_ over a region of interest chosen from the experimental velocity distribution. The evaluated χ^2^(τ) values are fitted quadratically as a function of τ to obtain the characteristic relaxation times. The evaluation of τ is carried out for each deflector current individually in order to be sensitive to any possible magnetic field dependence. For nondeflected clusters, the fitting procedure is not applicable, and we therefore report an upper limit on τ for such cases. The results are summarized in Table 1.

**Table 1:** Characteristic Spin Relaxation Times τ Obtained from Quadratic Fitting of χ^2^(τ) at *I*_d_ = 700 A^a^

	Na(H_2_O)*_n_*	Na(NH_3_)*_n_*	Na(MeOH)*_n_*	Na(DME)*_n_*
*n*	τ [μs]	τ [μs]	τ [μs]	τ [μs]
1	>τ = 100 ◆	>τ = 100 ◆	112(15) ◬	105(19) ◬
2	<25 ◇	92(12) ◬	<25 ◇	34(30) ◬
3	<25 ◇	72(12) ◬	<25 ◇	<25 ◇
4	<25 ◇	62(10) ◬	<25 ◇	

a The experimental deflection behavior is represented by the symbols. ◆: *m*_*s*_ = ±1/2 deflection, ◬: partial deflection, and ◇: nondeflected.

With the characteristic relaxation times τ, we determine
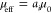
7where *a*_*i*_ is the unitless average attenuation factor of the exponential term for a single particle in eq 4 during the interaction time *t*_m_ while traversing the deflector, defined by the deflector length *s*_def_ and molecular beam velocity *v*_*i*_.
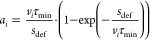
8

**Figure 9:**
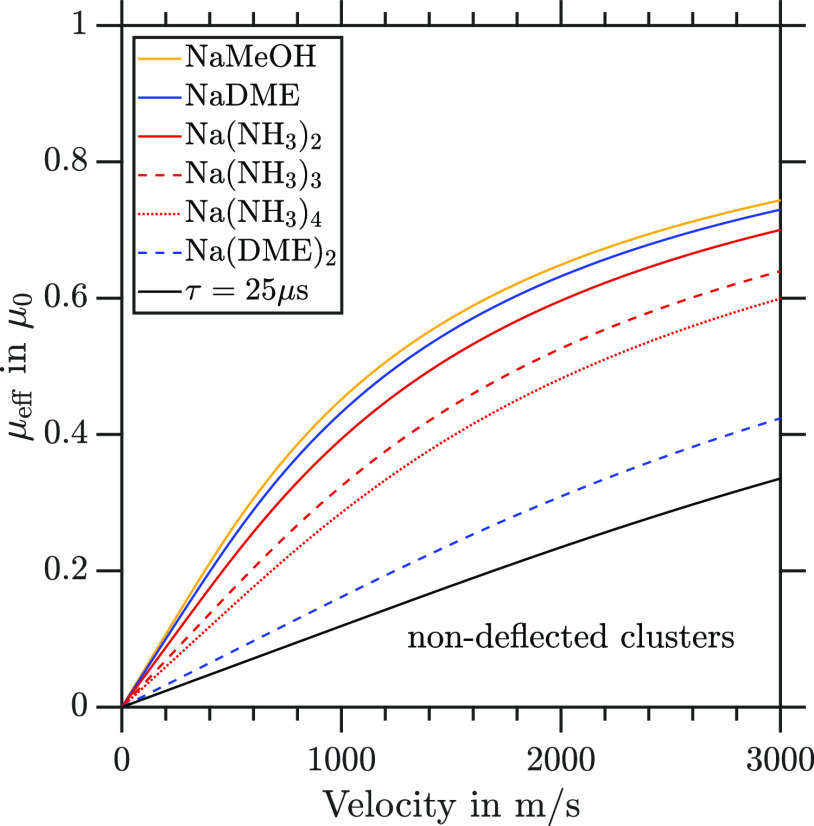
Effective magnetic moment μ_eff_ as a function of molecular beam velocity for the partially deflecting clusters and limiting values for the nondeflected clusters (black line).

In Figure 9, the effective magnetic moments for the partially deflecting clusters and the upper limit for the nondeflected clusters (black line) are shown as a function of the cluster velocity. Within our current experimental setup, we are not able to distinguish between clusters with effective magnetic moments smaller than the upper limit (black line in Figure 9) for a given molecular beam velocity. This black line represents the detection limit for deflection in our current experimental setup.

The interaction with the magnetic field gradient is defined by *v*_*i*_ and the deflector length dictates the observable μ_eff_ within the applied model. A comparison of different μ_eff_ values is therefore only meaningful when compared at the same velocity or interaction time with a magnetic field gradient. For a constant velocity *v*_*i*_ at any value (see Figure 9), we determine the following experimental deflection trend
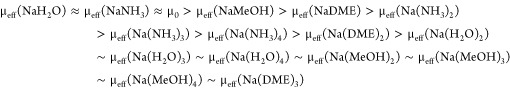


### 3.5 Discussion

The experimentally observed deflection trends can be rationalized by using the density and thermal population of rovibrational states within the Zeeman energy splitting. Harmonic frequency analysis of density functional theory (DFT)-optimized cluster geometries were carried out with the Gaussian program package.^50^ In a first step, the dispersion-corrected ωB97XD density functional with a 6-31+G* basis set was used to optimize cluster geometries. In the second step, harmonic frequencies and rigid rotor rotational constants were evaluated for various geometric isomers. In a last step, the converged minimal energy structures were reoptimized with MP2 calculations with an aug-cc-PVDZ basis set, and additional harmonic frequency calculations were carried out.

The Zeeman diagram of an *m*_*s*_ = ±1/2 system is split into two eigenstates according to their spin orientation. In the case of *m*_*s*_ = ±1/2, zero field splitting of spin microstates can be neglected as only one unpaired electron is present. Available rotational states increase the number of diabatic Zeeman-like levels to 2*J* + 1 = (2*S* + 1)·(2*R* + 1), where *S* is the spin, *R* the rotational, and *J* is the total angular momentum (neglecting contributions from additional angular momenta). If *S* and *R* are uncoupled, then degeneracies of the Zeeman-like states are allowed. However, if *S* and *R* are even weakly coupled, then crossings of the Zeeman-like states are avoided for adiabatic states with equal *J*. At an avoided crossing, *m*_*s*_ changes sign in both of the associated Zeeman-like states, while the total angular momentum *J* = *R* + *S* is conserved. For *m*_*s*_ = ±1/2 systems, this leads to Δ*m*_*s*_ = 1, which only allows for spin transitions between adiabatic Zeeman-like states with Δ*M*_R_ = 1. In terms of the avoided crossing model,^29^ clusters with a higher density of populated rovibrational states are expected to undergo more spin transitions and on average exhibit less deflection, when compared to clusters with lower densities of thermally accessible adiabatic Zeeman-like levels.

In order to discuss our experimental findings and relative deflection trends, we analyze rovibrational eigenstates *E*_*n*,*R*_. We choose to describe *E*_*n*,*R*_ with respect to the vibrational energies of a harmonic oscillator and the rotational energies of a rigid rotor.
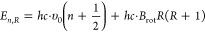
9where *n* is the vibrational quantum number, υ_0_ is the vibrational eigenfrequency, *R* is the rotational quantum number, and *B*_rot_ is the rotational constant. With estimated vibrational *T*_vib_ and rotational *T*_rot_ temperatures, we express the average number of thermally accessible rovibrational states in terms of the partition function. The density of rovibrational states is expressed as the average number of populated states within the maximum achievable Zeeman energy splitting Δ*E*_Zeeman_ = 2μ_B_*B*_max_ ≈ 2 cm^–1^, where μ_B_ is the Bohr magneton and *B*_max_ ≈ 2*T*. The following discussions are based on the assumption of thermally equilibrated clusters, where the population of states is described by a Maxwell–Boltzmann distribution at estimated thermal energies *k*_B_*T*.

In the case of *S* and *R*-coupled adiabatic Zeeman-like levels, states with *m*_*s*_ = ±1/2 contributions are able to undergo avoided crossings if neighboring states with Δ*M*_R_ = 1 have an energy difference smaller than the Zeeman energy splitting. We chose the maximal Zeeman energy splitting for a spin 1/2 system (Δ*E*_Zeeman_ ≈ 2 cm^–1^) as an upper limit for all investigated clusters. Harmonic frequency calculations of the investigated clusters and their structural isomers (see the SI) show that differences in vibrational energies of thermally accessible modes are larger than the Zeeman energy splitting. We find that rotational energy level differences, for a rigid rotor approximated by *E*_rot_ = ⟨*B*⟩_rot_*J*(*J* + 1) with the average rotational constant ⟨*B*⟩_rot_ = (*A*_rot_ + *B*_rot_ + *C*_rot_)/3 (see *A*, *B*, *C*, and ⟨*B*⟩_rot_ in Tables S2–S5), are less than Δ*E*_Zeeman_ for all studied clusters except NaH_2_O and NaNH_3_, which are the two clusters deflected as spin 1/2 particles. Thus, multiple rotational levels within Δ*E*_Zeeman_ are to be expected in the cases in which we measure reduced deflection. We take the number of avoided crossings a cluster undergoes to be governed by the density of rotational energy levels within Δ*E*_Zeeman_ and rather independent of the population of vibrational modes, as they are spaced by more than Δ*E*_Zeeman_. We, therefore, discuss avoided crossings in terms of rotational states, assuming no additional contributions from excited vibrational states.

#### 3.5.1 Average Number of Rotational States within Δ*E*_Zeeman_

We represent the average number of rotational eigenstates within Δ*E*_Zeeman_ through the rotational partition function *Q*_rot_. We use *Q*_rot_ to represent the average number of populated states. Then, *Q*_rot_ divided by the average rotational energy *k*_B_*T*_rot_ and multiplied by Δ*E*_Zeeman_ corresponds to the average number of states within Δ*E*_Zeeman_. Our analysis is based upon a DFT-optimized cluster with various isomers (ωB97XD/6-31+G*). In the high-temperature approximation, the rotational partition function of a nonlinear rotor is expressed by
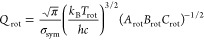
10where σ_sym_ is the symmetry number, *T*_rot_ the rotational temperature, and *A*_rot_, *B*_rot_, and *C*_rot_ are the rotational constants of the rigid rotor. Since the rotational temperatures of free clusters are not well-defined, we assume a temperature range 10 K ≤ *T*_rot_ ≤ 50 K. This range comes from the comparison of the vibrational and rotational energy transfer cross sections σ_vib_ and σ_rot_, which follow σ_vib_ ≪ σ_rot_.^51,52^^51,52^ In an adiabatic expansion, the differences in effective energy transfer lead to *T*_rot_ < *T*_vib_. Fuchs et al.^53^ determined *T*_rot_ < *T*_vib_ with 5 K ≤ *T*_rot_ ≤ 20 K for small metal clusters generated in a supersonic expansion at a nozzle temperature of 16 K. In our experiments, nozzle temperatures are significantly higher (10 °C ≤ *T*_nozzle_ ≤ 150 °C) and collisions with sodium atoms in the oven are expected to affect the cluster temperatures. Although it is unclear how much this influences the cluster temperatures, as the cluster can evaporate monomer units to dissipate the acquired energy, we take a rotational temperature range of 10 K ≤ *T*_rot_ ≤ 50 K for the analysis.

**Figure 10:**
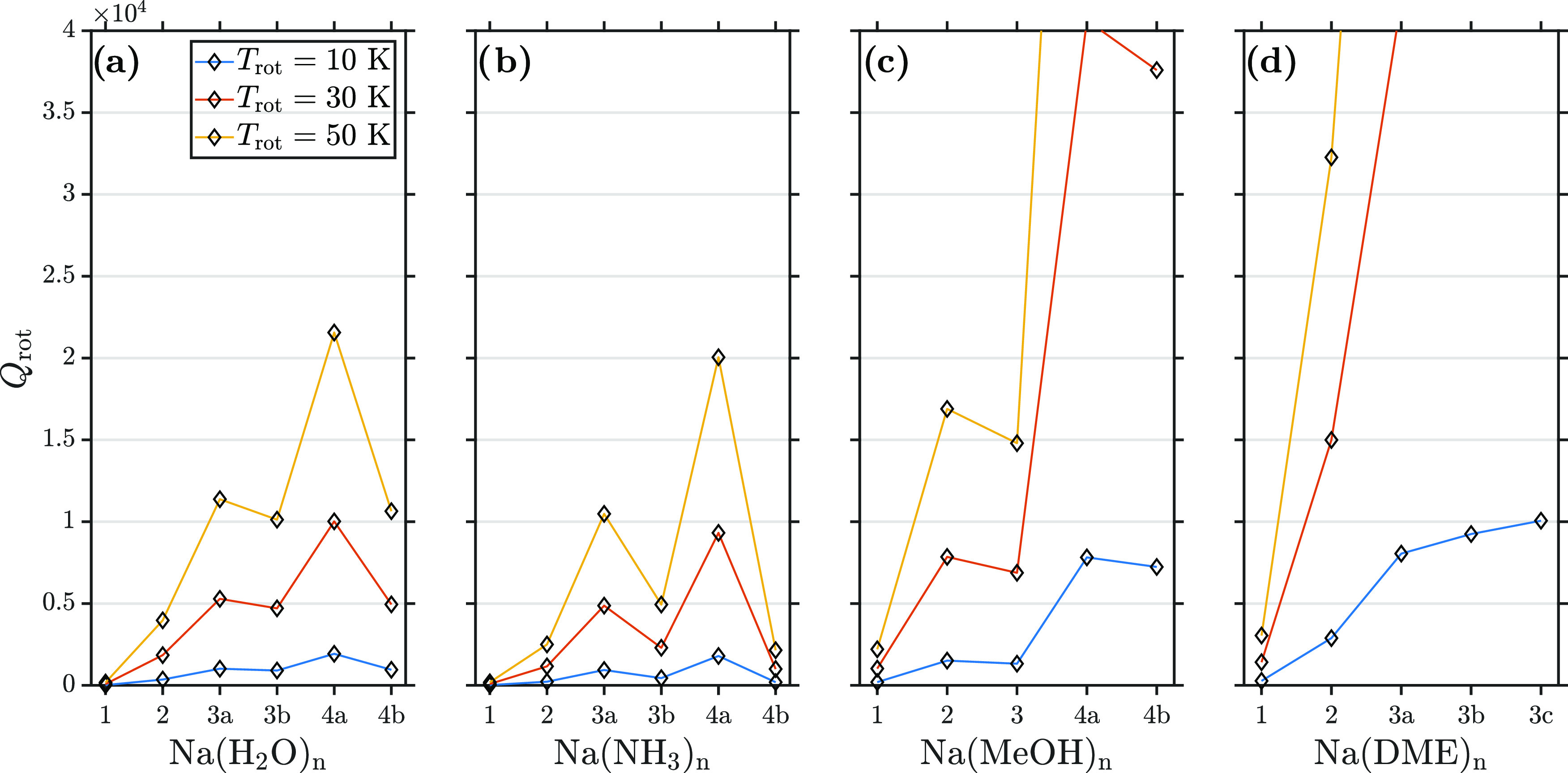
Rotational partition function *Q*_rot_ of (a) Na(H_2_O)*_n_*, (b) Na(NH_3_)*_n_*, (c) Na(MeOH)*_n_*, and (d) Na(DME)*_n_* for various rotational temperatures *T*_rot_. The clusters are evaluated from *n* = 1 to 4 (*n* = 3 for Na(DME)*_n_*). Letters (e.g., “a” and “b”) denote isomers of a given cluster size. The different structures are described in Tables S2–S5, and geometries are shown in the SI.

With estimated rotational temperatures and rotational constants, we determine the rotational partition functions *Q*_rot_ via eq 10. The results are shown in Figure 10 for 1 ≤ *n* ≤ 4 where the letters (e.g., a and b) refer to different isomers of a cluster with a given number of monomer units (*n*) (see Figures S3–S6 in the SI). This analysis was performed to see if the average number of rotational states within Δ*E*_Zeeman_ (represented by *Q*_rot_) correlated with the trend in the magnetic deflection behavior we measured in this study. If so, we should find that an increase in thermally accessible rotational states coincides with a reduction in deflection.^29,38−41,47^

Comparison of *Q*_rot_ in the cluster systems in the temperature range 10 K ≤ *T*_rot_ ≤ 50 K shows the lowest values for NaH_2_O and NaNH_3_. Furthermore, the rotational state population is only weakly dependent on rotational temperature for NaH_2_O and NaNH_3_. This is consistent with our deflection measurements having a similar behavior to that of a spin 1/2 particle or an effective magnetic moment similar to μ_0_. NaMeOH and NaDME exhibit a similarly low *Q*_rot_ at 10 K but a more pronounced increase of *Q*_rot_ with temperature and experimentally only reduced deflection compared to a simulated spin 1/2 particle of the same mass.

Estimates of thermal temperatures showed that Na(DME)*_n_* clusters are expected to be colder than the other clusters studied due to the relatively low nozzle temperature used for the DME expansion (see Table S1) and relatively weak intermolecular forces binding DME molecules. With *T*_rot_(Na(NH_3_)*_n_*) > *T*_rot_(Na(DME)*_n_*), we estimate fewer populated rotational states for NaDME than for Na(NH_3_)_2_ and confirm the experimental observation of μ_eff_(NaDME) > μ_eff_(Na(NH_3_)_2_). Slight deflection of Na(DME)_2_ is consistent with it having a lower rotational temperature, whereas Na(MeOH)_2_ shows an increase of populated rotational states, which could explain its lack of deflection. At the lowest temperatures considered here, the change in the rotational state density between NaH_2_O and Na(H_2_O)_2_ is unlikely to be the reason for the absence of deflection for Na(H_2_O)_2_. This leads us to the idea that the Na(H_2_O)*_n_* clusters may be hotter than the other sodium-doped solvent clusters studied.

The larger clusters of Na(MeOH)_3,4_ and Na(DME)_3_ exhibit significant population in many rotational states, which supports the observation of no significant deflection. The absence of deflection for Na(H_2_O)_3,4_ is difficult to explain by only comparing thermally accessible rotational states. Possible explanations include a higher rotational temperature or contributions other than spin–rotational couplings. The structural isomers of Na(NH_3_)_3,4_ show substantially different populations of rotational states. For Na(NH_3_)_3_, isomer 3b is 83 meV lower in energy than isomer 3a. The experimental observation of partial deflection for Na(NH_3_)_3_ may imply a preferential production of isomer 3b and its lower rotational state density. Similar arguments hold for Na(NH_3_)_4_ which shows partial deflection, which may be explained by preferential production of the tetrahedral isomer 4b with its significantly reduced population of rotational states compared to isomer 4a. Previous work of our group^10^ found *n* = 4 to be a magic number for the photoelectron anisotropy in sodium-doped ammonia clusters. These complementary experiments support the idea that Na(NH_3_)_4_ is produced and deflects a highly symmetric tetrahedral structure with substantially lower rotational state density. For the *T*_*d*_ structure σ_sym_ = 12, less symmetric isomers with lower σ_sym_ (IB in ref (10)) would cause an increase in rotational state density and may lead to reduced deflection. This hints at the possibility of cluster-symmetry selective deflection measurements. With a combination of magnetic deflection of neutral clusters and angle-resolved photoelectron spectroscopy, one could characterize isomer-dependent magnetic and electronic properties. In such an experiment, magnetic deflection of highly symmetric cluster structures could be demonstrated by a decrease in the photoelectron anisotropy parameter β (measured in the nondeflected cluster beam), which can be related to the orbital angular momentum of the electron ejected upon photoionization.^10,11,54^

In general, we find a correlation between experimental deflection trends and rotational level spacings and populations. The most significant deviations of the experiment and model are possibly due to differences in cluster rotational temperatures, with a proposed order in our experiments being *T*_rot_(Na(DME)*_n_*) < *T*_rot_(Na(NH_3_)*_n_*) < *T*_rot_(Na(MeOH)*_n_*) < *T*_rot_(Na(H_2_O)*_n_*). Although influences from contributions other than spin–rotational coupling may also be important, further and more refined studies are needed.

## 4 Conclusions

The magnetic deflection behavior of Na(H_2_O)*_n_* (*n* = 1–4), Na(NH_3_)*_n_* (*n* = 1–4), Na(MeOH)*_n_* (*n* = 1–4), and Na(DME)*_n_* (*n* = 1–3) clusters was characterized. We showed that the fully deflecting *m*_*s*_ = ±1/2 character of NaH_2_O and NaNH_3_ is consistent with these clusters having large rotational level splitting compared with Δ*E*_Zeeman_. In all other clusters studied, the observation of reduced deflection was found to be consistent with an increased population of closely spaced, excited rovibrational states. Magnetic deflection observed to be less than that of a free *m*_*s*_ = ±1/2 system was modeled by the attenuation of the magnetic moment μ_0_. With this approach, we were able to retrieve characteristic spin relaxation times τ. The relative experimental deflection trends were discussed in terms of thermally accessible rovibrational states in an attempt to understand trends in their deflection behavior. Thermal energies of the studied clusters were estimated using evaporative ensemble theory and quantum chemical calculations of vaporization enthalpies. The rovibrational states were analyzed in the harmonic oscillator and rigid rotor approximations of geometry-optimized cluster geometries.

The magnetic deflection behavior observed in the weakly bound cluster systems studied here does not have an obvious explanation and will need further investigations to give a clear understanding. For the moment, we point to spin–rotational couplings and avoided crossings as a possible cause for a reduction in the effective magnetic moment and reduced deflection. More refined experimental and theoretical work is needed in order to understand other contributions to the attenuation of the magnetic moment while traversing a magnetic field gradient. In particular, well-defined and low temperatures for vibrational *T*_vib_ and rotational *T*_rot_ degrees of freedom would simplify the interpretation of measured trends in magnetic deflection. Additional contributions, such as spin–orbit and hyperfine couplings, may perturb the Zeeman-like levels and influence the deflection behavior. Methods to calculate the cluster Zeeman effect with further angular momenta contributions have been summarized by Sears.^55^ Berdyugina and Kuzmychov^56^ determined the energy level structure of CrH in the presence of a magnetic field via quantum chemical calculations and predicted magnetic deflection behavior. A similar approach seems feasible for small clusters (e.g., NaNH_3_ and NaH_2_O); however, for larger systems, calculations will be challenging. Calculations of the Zeeman diagram for small sodium-doped clusters are of interest to evaluate contributions from other angular momenta in magnetic deflection experiments.

## Supporting Information Available

Cluster generation conditions; explanation of magnetic field pulses; velocity dependence of magnetic deflection; calculated cluster geometries, symmetries and rotational constants; vibrational level analysis of studied clusters; and population of vibrational levels as a function of temperature (PDF)
