# Supplementary material for: Comparison of Magnetic Deflection among Neutral Sodium-Doped Clusters: Na(H2O)n, Na(NH3)n, Na(MeOH)n, and Na(DME)n
Source: J Phys Chem A. 2023 Oct 5;127(41):8544–55. doi: 10.1021/acs.jpca.3c03820 (PMC10591511; doi:10.1021/acs.jpca.3c03820)
Supplement: Supplementary file 1 — jp3c03820_si_001.pdf [file jp3c03820_si_001.pdf]

Supporting Information:

Comparison of Magnetic Deflection among Neutral Sodium-doped Clusters: $Na(H_2O)_n$, $Na(NH_3)_n$, $Na(MeOH)_n$ and $Na(DME)_n$

Jonathan V. Barnes, Dominique P. Borgeaud dit Avocat, Edith Simmen, Huanyu Yang, Bruce L. Yoder and Ruth Signorell*

ETH Zürich, Department of Chemistry and Applied Biosciences, Zürich 8093, Switzerland

* To whom correspondence should be addressed. E-mail: rsignorell@ethz.ch

Cluster beams

Molecular clusters presented in this work were generated via supersonic expansion of either neat gas or mixtures into vacuum. The cluster formation conditions of the individual substances are summarized in Table S1. H_2O clusters were generated with a pulsed valve, while all other cluster beams were generated via continuous supersonic expansions.

Table S1. Cluster generation conditions for the investigated sodium-doped solvent clusters.

Gas mixture	p [bar]	d_{nozzle} [µm]	$T_{bubbler}$ [ºC]	T_{nozzle} [ºC]	T_{oven} [ºC]
DME (neat)	3.5	55	-	10	195
He/H_2O (7:1)	8.0	150	105	140	195
N_2/H_2O (1.5:1)	6.0	150	145	160	195
MeOH (neat)	1.3	50	70	100	230
NH_3 (neat)	6.0	35	-	115	195

Magnetic field gradient of deflector

Our magnetic field gradient is given by

$$\nabla B(r,t,I) = \nabla\big(\mathrm{b(r)}\, p(t)\, f_B(I)\big) = \nabla \mathrm{b(r)}\, p(t)\, f_B(I) \qquad \text{(S1)}$$

where $\nabla\mathrm{b(r)}$ denotes the magnetic field gradient for a constant current of 1000 A (see Fig. S1A), $p(t)$ is the pulse profile of our magnetic field (see Fig. S1B), and $f_B(I)$ is the scaling factor for different currents applied to the deflector coils (see Fig. S1C).

The gradient of the magnetic field, shown in Figure S1A, was obtained from a COMSOL simulation assuming a 2D model of the deflector cross section and a DC current of 1000 A.

The time dependence of the magnetic field pulse produced by individual coils in our deflector was measured using a Hall probe. Temporal profiles of pulses for the different currents are very similar and therefore the pulse obtained with a current of 1000 A is used as a reference. For the simulations, the scaling factor $f_B(I)$ is introduced to model the different currents used in the experiment.

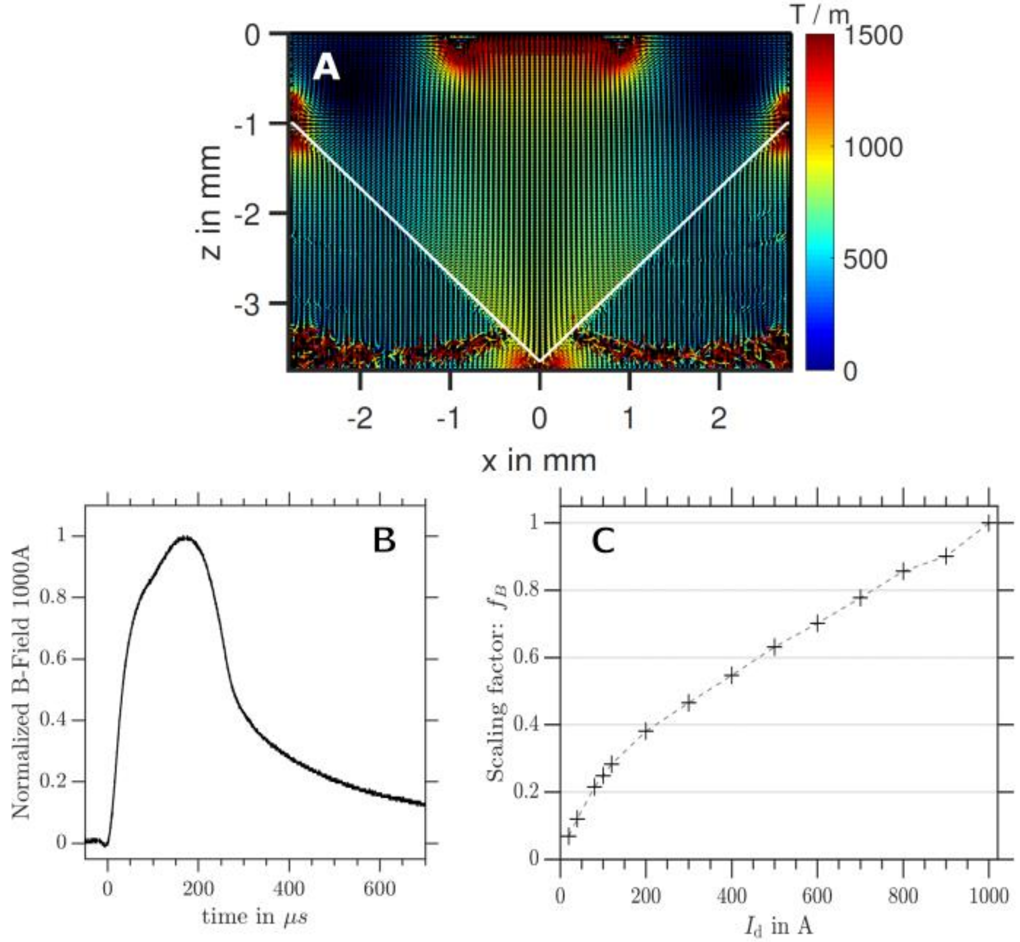

Figure S1. Overview of the magnetic field gradient components. A) Gradient along the x and z (deflection) axis is shown. The white diagonal lines along with a horizontal line at z=0 define the open channel of our deflector. B) The pulse shape of the magnetic field is shown for 1000 A. C) Current specific scaling factor f_B of the magnetic field. See Barnes et al.[1] for more details.

NaH_2O: Velocity dependence of magnetic deflection

Figure S2 displays velocity distributions for deflector 'off' and 'on' measurements with I_d = 500 A and three relative time delays $\Delta t_{L\text{-}v}$ = 1.30 ms, 0.80 ms and 0.70 ms. Mechanical recoil of the EL-valve's plunger produces several opening of the valve for each trigger pulse, such that multiple gas pulses are generated. This effect of the plunger recoil is most visible in the deflector 'off' trace at $\Delta t_{L\text{-}v}$ = 1.30 ms, which exhibits four distinct velocity profiles in the range of 1100 – 1800 m/s. We took advantage of this mechanical characteristic, as it allows us to probe a broad velocity range by varying $\Delta t_{L\text{-}v}$. Sampling faster velocities requires shorter relative time delays, as the particles of interest reach the ionization region quicker. This is illustrated for $\Delta t_{L\text{-}v}$ = 0.80 ms (mid panel) with velocities 1500 – 2300 m/s. Even faster clusters (1800 – 2500 m/s) are sampled at $\Delta t_{l\text{-}v}$ = 0.70 ms (bottom panel). These clusters represent the 'fastest' velocity distribution of NaH_2O. For $m_s = \pm 1/2$ particles, deflection magnitude is expected to increase with slower velocities, since slower particles experience magnetic field gradient for a longer time as they traverse the deflector. So, we would expect an increase in γ_d with decreasing velocity of the cluster beam. Clear experimental evidence of this trend can be seen in Figure S2 (top panel) for the deflector 'on' trace (green dots) which exhibits nearly full deflection ($\theta_{rel} \approx 0$) for velocities < 1500 m/s. As seen in all panels of Figure S2, residual signals from incomplete deflection are observed for velocities > 1600 m/s. With $\theta_{rel} \approx 0.7$ for the fastest (~2300 m/s) sampled NaH_2O clusters.

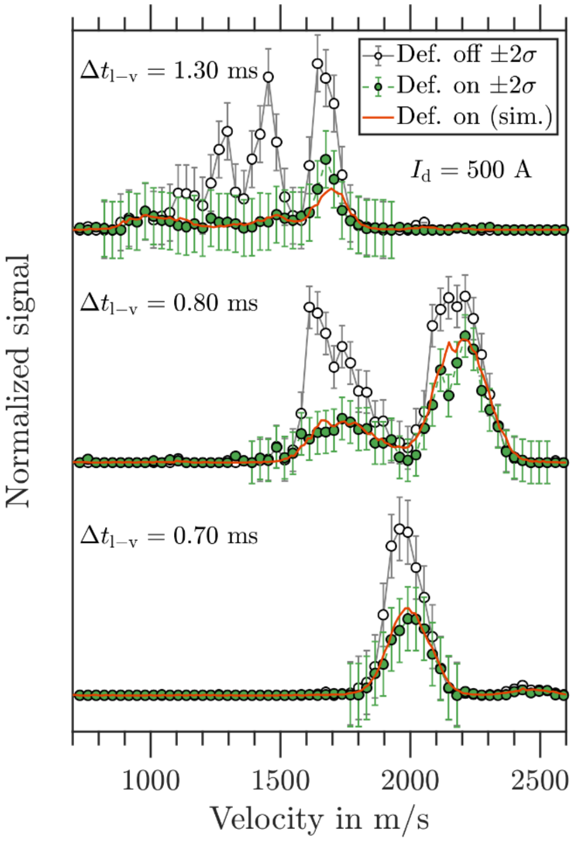

Figure S2. Experimental NaH_2O velocity distributions retrieved from photoion VMIs (10 000 laser shots per VMI) for deflector 'off' (black circles with 2σ) and deflector 'on' (green dots with 2σ) with I_d = 500A and the corresponding time delays: $\Delta t_{l\text{-}v}$ = 1.30 ms (top panel), $\Delta t_{l\text{-}v}$ = 0.80 ms (middle panel) and $\Delta t_{l\text{-}v}$ = 0.70ms (bottom panel). The corresponding simulated NaH_2O velocity distributions for m_s = ±½ particles, obtained by combining MD and SIMION simulations are shown as red traces.

Cluster Geometries

Figures S3-S6 show the cluster geometries referred to in Figure 10 and Tables S2-S5.

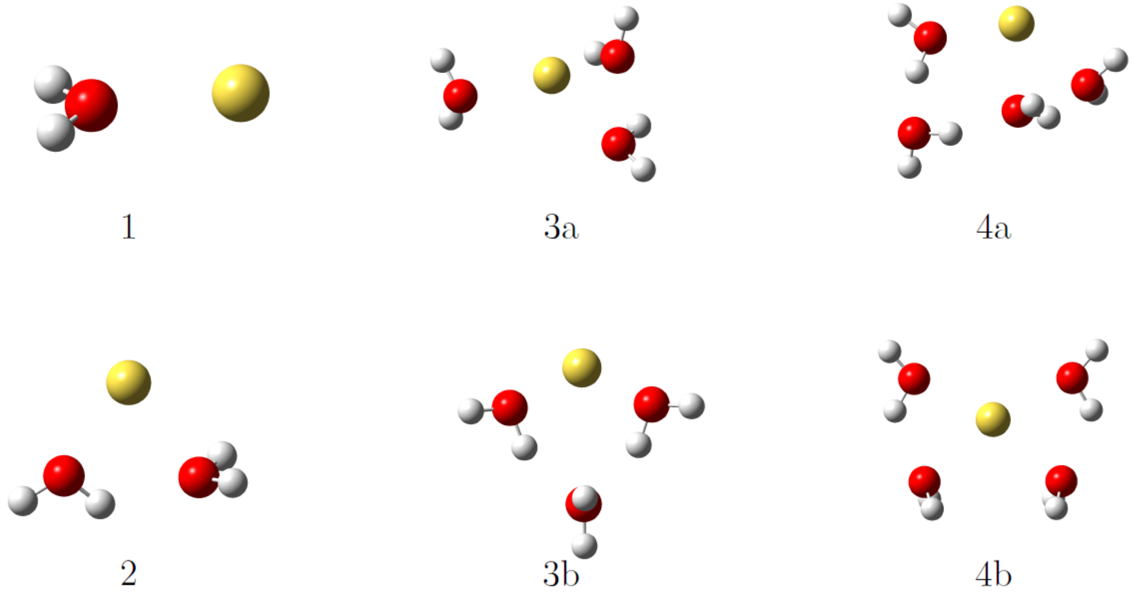

Figure S3. DFT optimized (ωB97XD/6-31+G*) cluster structures of $Na(H_2O)_n$.

Table S2. $Na(H_2O)_n$: Rotational constants of DFT optimized cluster structures (ωB97XD/6-31+G*) with their point group (PG), symmetry number and energy difference between structural isomers ΔE_{iso}.

$Na(H_2O)_n$	PG	σ_{sym}	A [cm^{-1}]	B [cm^{-1}]	C [cm^{-1}]	$\langle B \rangle_{rot}$ [cm^{-1}]	ΔE_{iso} [meV]
1	C_{2v}	2	13.94	0.28	0.28	4.83	-
2	C_s	1	0.29	0.22	0.13	0.21	-
3a	C_1	1	0.19	0.08	0.06	0.11	+ 64
3b	C_s	1	0.13	0.12	0.08	0.11	0
4a	C_1	1	0.10	0.06	0.04	0.07	0
4b	C_{2v}	2	0.12	0.06	0.04	0.07	+ 107

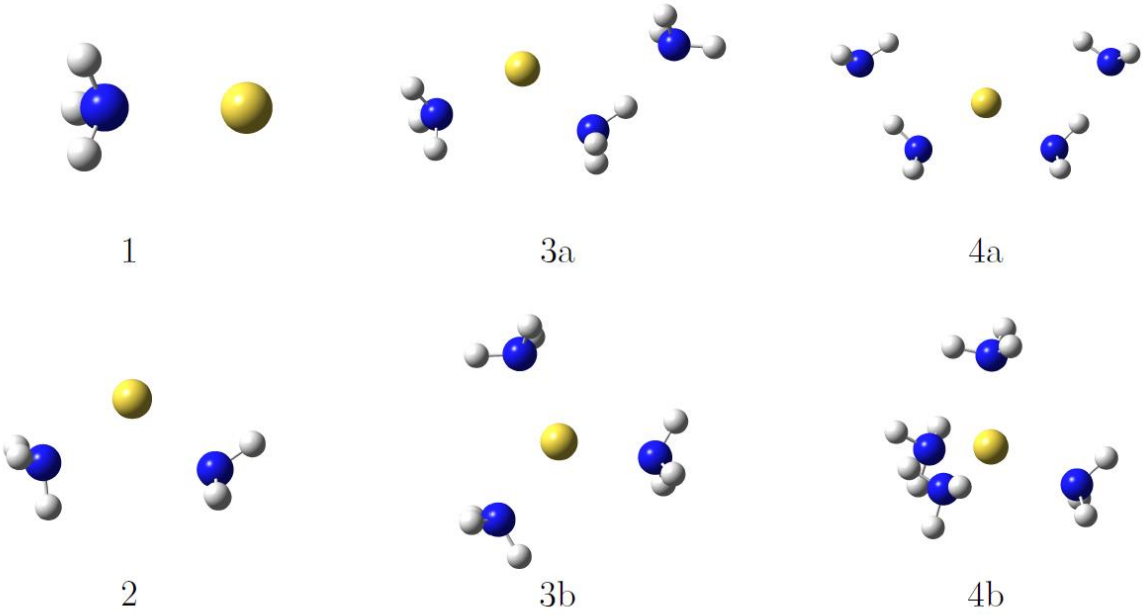

Figure S4. DFT optimized (ωB97XD/6-31+G*) cluster structures of $Na(NH_3)_n$.

Table S3. $Na(NH_3)_n$: Rotational constants of DFT optimized cluster structures (ωB97XD/6-31+G*) with their point group (PG), symmetry number and energy difference between structural isomers ΔE_{iso}.

$Na(NH_3)_n$	PG	σ_{sym}	A [cm^{-1}]	B [cm^{-1}]	C [cm^{-1}]	$\langle B \rangle_{rot}$ [cm^{-1}]	ΔE_{iso} [meV]
1	C_{3v}	3	6.25	0.26	0.26	2.26	-
2	C_{2v}	2	0.45	0.12	0.10	0.22	-
3a	C_1	1	0.65	0.05	0.04	0.25	+ 83
3b	C_3	3	0.11	0.10	0.06	0.09	0
4a	C_{2v}	2	0.13	0.03	0.02	0.06	+ 235
4b	T_d	12	0.06	0.06	0.06	0.06	0

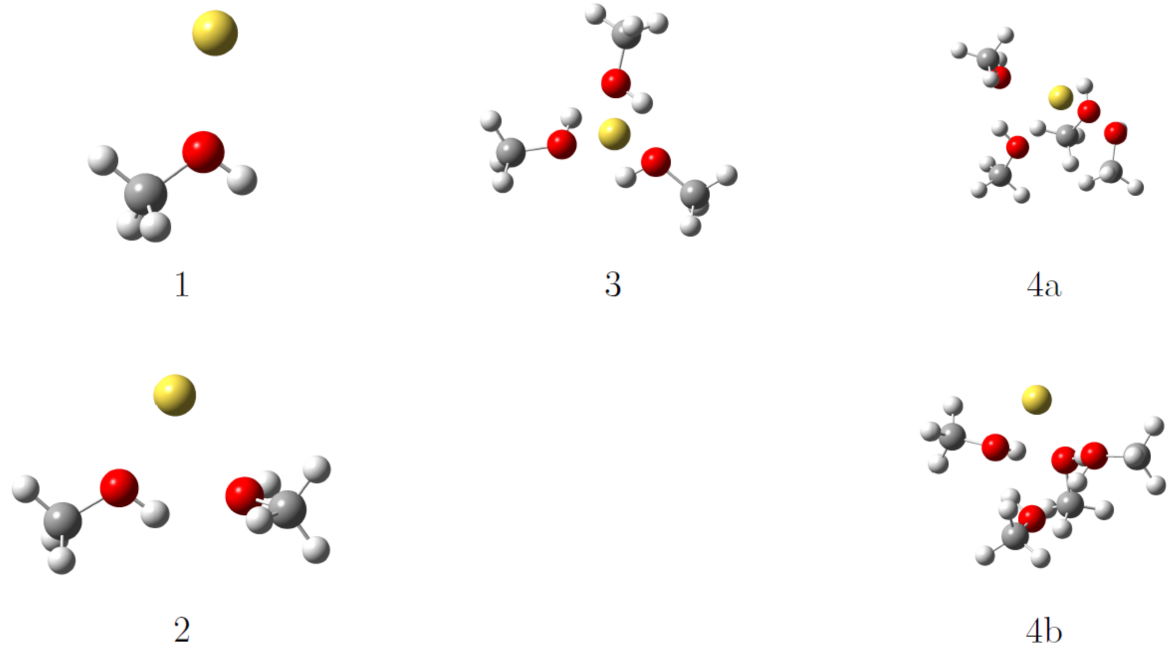

Figure S5. DFT optimized (ωB97XD/6-31+G*) cluster structures of $Na(MeOH)_n$.

Table S4. $Na(MeOH)_n$: Rotational constants of DFT optimized cluster structures (ωB97XD/6-31+G*) with their point group (PG), symmetry number and energy difference between structural isomers ΔE_{iso}.

$Na(MeOH)_n$	PG	σ_{sym}	A [cm^{-1}]	B [cm^{-1}]	C [cm^{-1}]	$\langle B \rangle_{rot}$ [cm^{-1}]	ΔE_{iso} [meV]
1	C_s	1	1.57	0.14	0.13	0.61	
2	C_1	1	0.15	0.06	0.05	0.09	
3	C_3	3	0.05	0.05	0.03	0.04	
4a	C_1	1	0.04	0.02	0.02	0.03	+ 351
4b	C_1	1	0.03	0.03	0.02	0.03	0

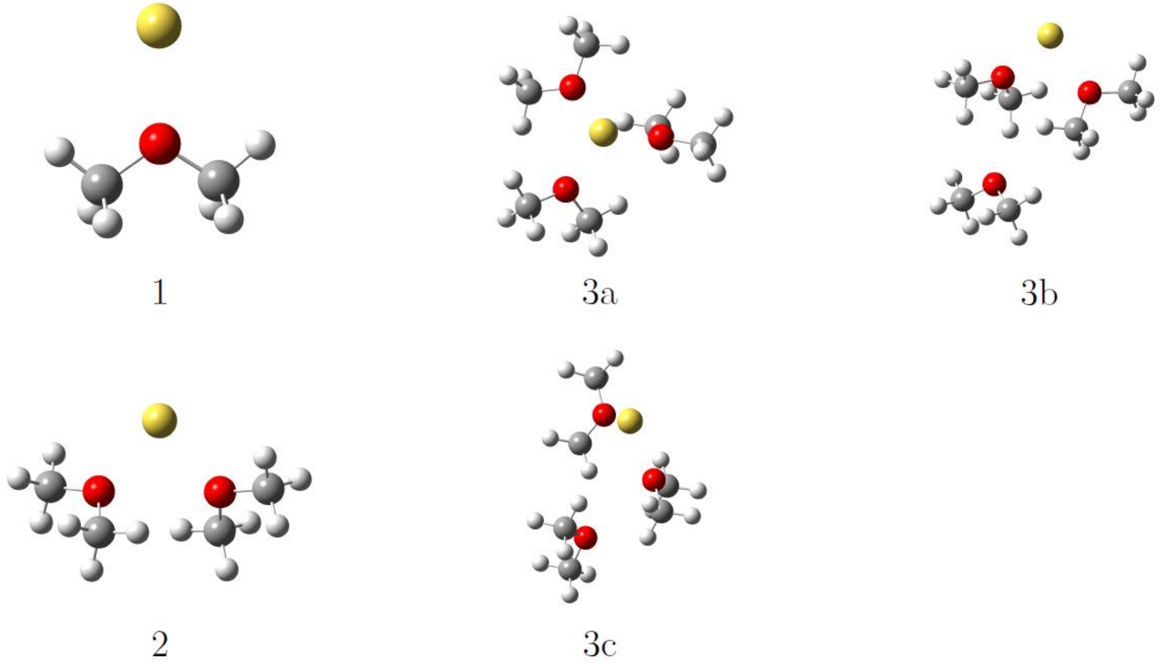

Figure S6. DFT optimized (ωB97XD/6-31+G*) cluster structures of $Na(MeOH)_n$.

Table S5. $Na(DME)_n$: Rotational constants of DFT optimized cluster structures(ωB97XD/6-31+G*) with their point group (PG), symmetry number and energy difference between structural isomers ΔE_{iso}.

**$Na(DME)_n$**	**PG**	**σ_{sym}**	**A [cm^{-1}]**	**B [cm^{-1}]**	**C [cm^{-1}]**	**$\langle B \rangle_{rot}$ [cm^{-1}]**	**ΔE_{iso} [meV]**
1	C_{2v}	2	0.33	0.12	0.09	0.18	
2	C_1	1	0.10	0.04	0.03	0.06	
3a	C_1	1	0.03	0.03	0.02	0.03	0
3b	C_1	1	0.04	0.02	0.02	0.03	+ 79
3c	C_1	1	0.04	0.02	0.01	0.02	+ 113

Vibrational level analysis

$Na(H_2O)_n$:

The lowest frequency vibration in the weakly bound non-covalent NaH_2O cluster is an out-of-plane wagging mode at 119 cm^{-1}. For the larger cluster $Na(H_2O)_2$, further vibrations well below 200 cm^{-1} occur. The lowest vibrational frequency in $Na(H_2O)_2$ is a Na–O stretching mode with 97 cm^{-1}, and additional hindered rotations of the water molecules at 109 cm^{-1}, 159 cm^{-1} and 168 cm^{-1}. For the

cluster sizes $Na(H_2O)_3$ and $Na(H_2O)_4$, the number of low-frequency vibrations increases further. $Na(H_2O)_3$ exhibits an O–O bending mode at 40 cm^{-1}, two Na–O stretching modes at 62 cm^{-1} and 83 cm^{-1} as well as three further hindered internal H_2O rotations < 200 cm^{-1}.

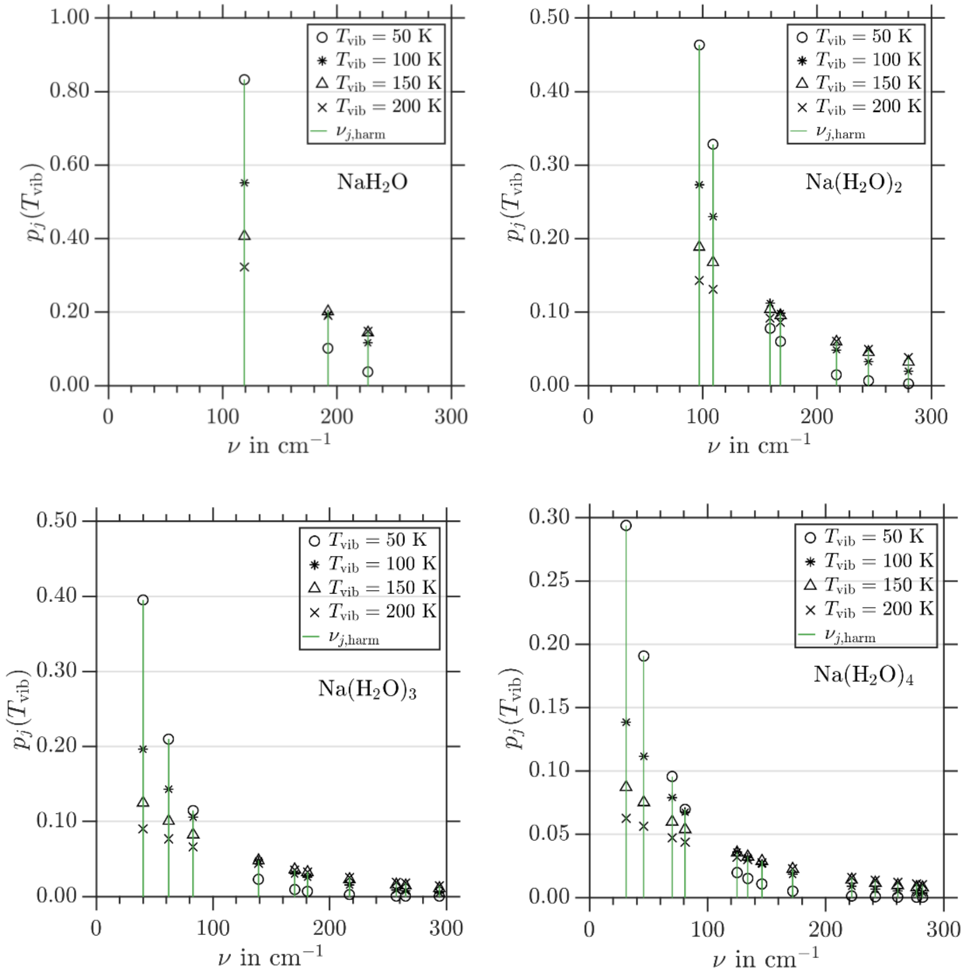

Figure S7. Calculated harmonic frequencies (MP2/aug-cc-PVDZ) for $Na(H_2O)_n$ clusters are shown as green vertical lines for NaH_2O, $Na(H_2O)_2$, $Na(H_2O)_3$ and $Na(H_2O)_4$ with ν < 300cm^{-1}. Population factors $p_j(T_{vib})$ are plotted for estimated cluster temperatures T_{vib}.

Na(NH$_3$)$_n$:

NaNH$_3$ exhibits a Na–N stretching mode at 197 cm^{-1} and a degenerate Na–N bending mode at 276 cm^{-1}. These vibrations show populations on the order of 20% for vibrational temperatures T$_{vib}$ > 50K. For the larger clusters Na(NH$_3$)$_{2–4}$, new types of low-frequency modes arise, with internal rotations of NH$_3$ around the Na–N axis and the N–Na–N bending vibration well below 100 cm^{-1}. As for these larger clusters, we predict the significant relative population of their vibrational modes at estimated vibrational temperatures (Figure S8).

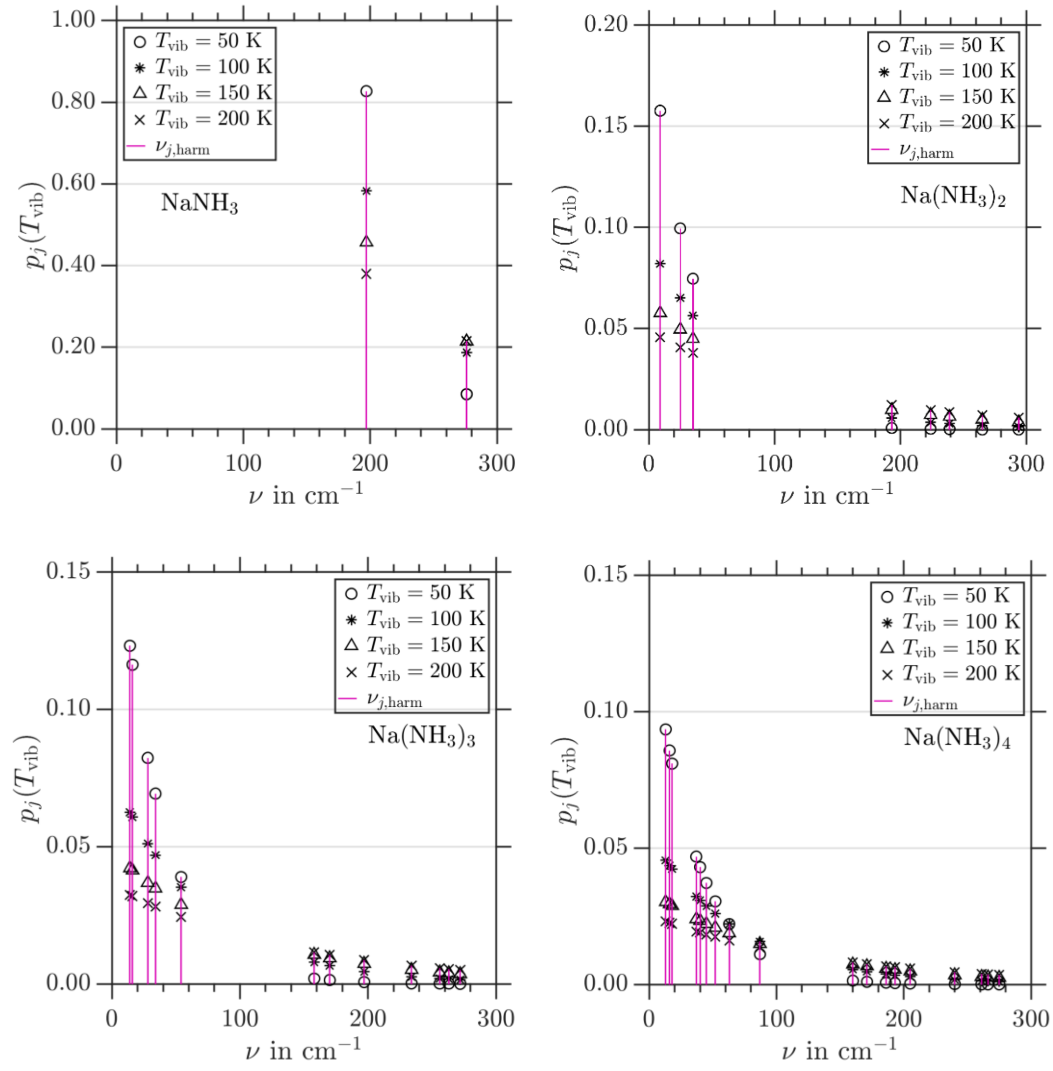

Figure S8. Calculated harmonic frequencies (MP2/aug-cc-PVDZ) for Na(NH$_3$)$_n$ clusters are shown as green vertical lines for NaNH$_3$, Na(NH$_3$)$_2$, Na(NH$_3$)$_3$ and Na(NH$_3$)$_4$ with ν < 300 cm^{-1}. Population factors $p_j(T_{vib})$ are plotted for estimated cluster temperatures T_{vib}.

$Na(MeOH)_n$:

NaMeOH exhibits a hindered rotation at 49 cm^{-1} and a wagging mode at 94 cm^{-1}. The O–Na stretching mode exhibits a frequency of 180 cm^{-1}. In the temperature range 50K < T_{vib} < 200K, we expect a significant population of these vibrational modes. For $Na(MeOH)_2$ additional accessible vibrational modes with energies well below 200 cm^{-1} are introduced by the addition of a second MeOH molecule. For $Na(MeOH)_3$ and $Na(MeOH)_4$ further thermally accessible intermolecular vibrational modes are established (Figure S9).

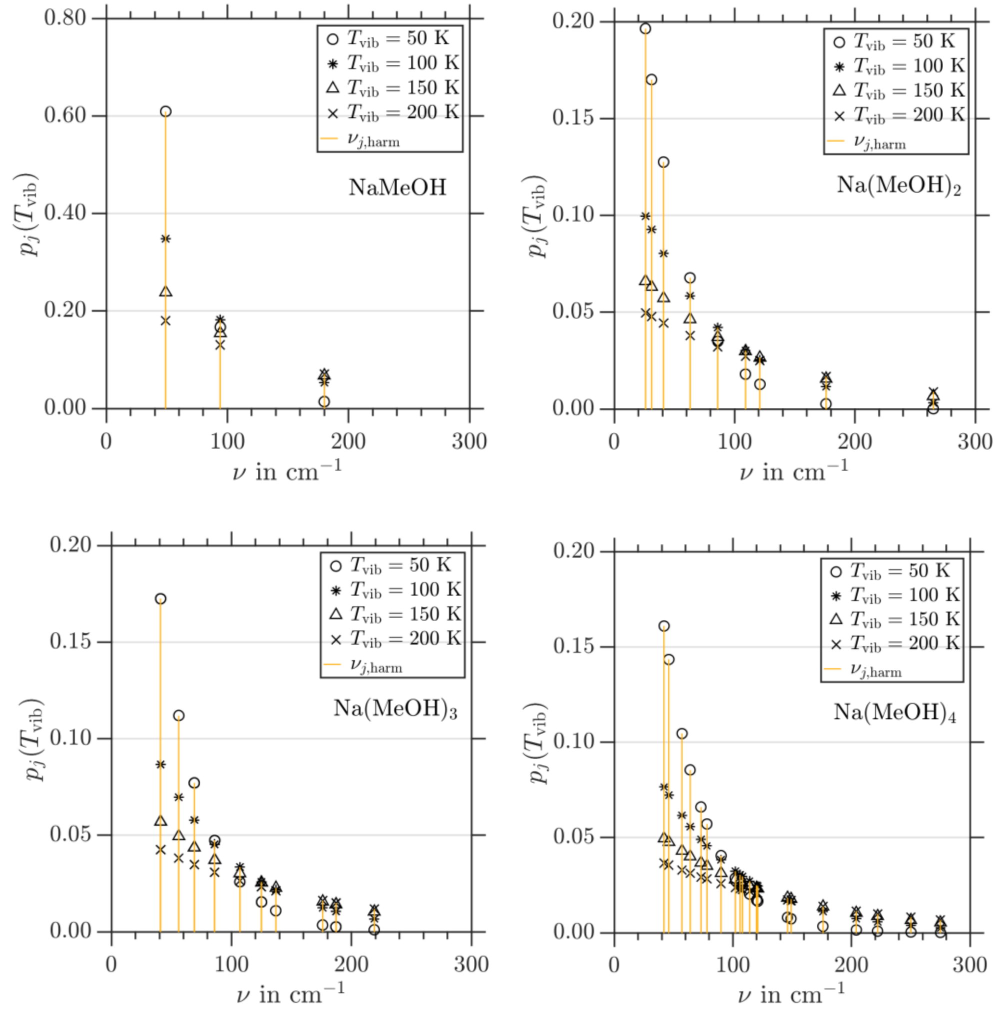

Figure S9. Calculated harmonic frequencies (MP2/aug-cc-PVDZ) for $Na(MeOH)_n$ clusters are shown as green vertical lines for NaMeOH, $Na(MeOH)_2$, $Na(MeOH)_3$ and $Na(MeOH)_4$ with ν < 300 cm^{-1}. Population factors $p_j(T_{vib})$ are plotted for estimated cluster temperatures T_{vib}.

Na(DME)ₙ:

For NaDME, hindered rotations are present with calculated vibrational energies of 51 cm^{-1} and 89 cm^{-1} and the Na–O stretching mode with 143 cm^{-1} (seen in Fig. 6.3.5a). For the larger cluster sizes n = 2, 3 additional types of low-frequency modes arise with DME–DME wagging and bending modes < 120 cm^{-1} (seen in 6.3.5c). For T_{vib} = 50K, the ground vibrational states and higher modes with < 100 cm^{-1} are especially highly populated (Figure S10).

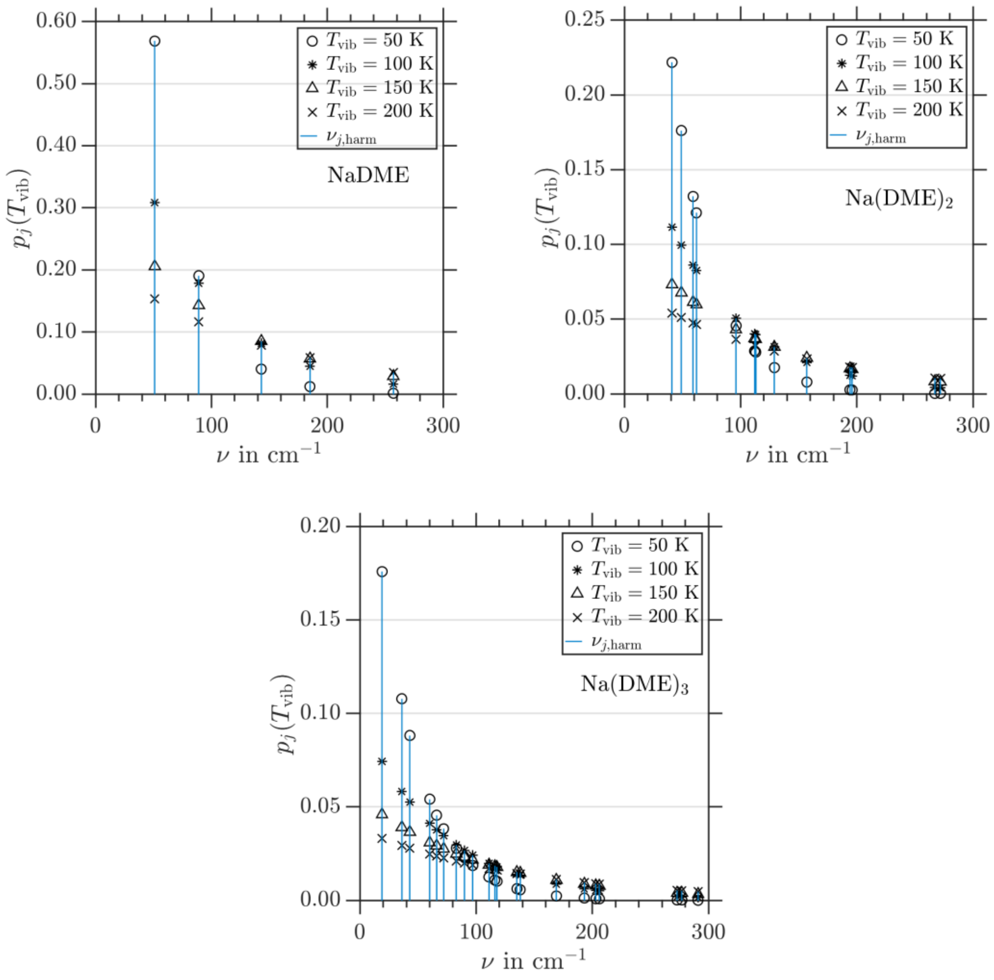

Figure S10. Calculated harmonic frequencies (MP2/aug-cc-PVDZ) for Na(DME)$_n$ clusters are shown as green vertical lines for NaMeOH, Na(MeOH)$_2$ and Na(MeOH)$_3$ with ν < 300 cm^{-1}. Population factors $p_j(T_{\mathrm{vib}})$ are plotted for estimated cluster temperatures T_{vib}.

References:

1. Barnes, J. V.; Beck, M.; Hartweg, S.; Luski, A.; Yoder, B. L.; Narevicius, J.; Narevicius, E.Signorell, R. Magnetic deflection of neutral sodium-doped ammonia clusters. *Phys. Chem. Chem. Phys.*, **2021**, *23,* 846-858.
